# Middle-range theory of the diagnosis of low situational self-esteem in undergraduate nursing students*

**DOI:** 10.1590/1518-8345.7662.4650

**Published:** 2025-08-18

**Authors:** Laís Vasconcelos Santos, Layane Ryane da Silva Souza, Marcos Manoel Sousa Silva, Marcos Venícios de Oliveira Lopes, Ana Luisa Brandão de Carvalho Lira

**Affiliations:** 1 Universidade Federal do Rio Grande do Norte, Natal, RN, Brazil.; 2 Universidade Federal do Ceará, Fortaleza, CE, Brazil.; 3 Scholarship holder at the Conselho Nacional de Desenvolvimento Científico e Tecnológico (CNPq), Brazil.

**Keywords:** Nursing Theory, Self Concept, Students, Nursing, Nursing Diagnosis, Nursing Research, Nursing Process

## Abstract

to elaborate a Middle-Range Theory for the diagnosis of situational low self-esteem in nursing students based on the mapping of scientific literature.

methodological study aimed at building a Middle-Range Theory, developed on the basis of the causal-theoretical model and based on Roy’s Adaptation Model. The process was operationalized in six stages: (1) defining the approach to building the theory; (2) defining the theoretical-conceptual models; (3) identifying the key concepts; (4) drawing up a pictorial scheme; (5) formulating propositions; and (6) establishing causal relationships and evidence for practice.

five essential attributes, 15 antecedents and 23 clinical consequents were identified. The analysis of these elements enabled the development of the Middle-Range Theory, consisting of an illustrative diagram, eight propositions, 12 causal relationships and evidence for practice.

the small number of studies shows how little the subject has been explored in national and international contexts. The Middle-Range Theory broadened the understanding of the diagnosis of situational low self-esteem, offering support for nurses’ work in the university context.

## Highlights

**(1)** MRT introduces elements for nurses to use in clinical practice. **(2)** Broadens understanding of the diagnosis of situational low self-esteem. **(3)** Supports the advancement of practice and research related to the student care process. **(4)** Can serve as a guiding resource for implementing actions to address low self-esteem.

## Introduction

The nursing diagnosis *Low situational self-esteem* from the NANDA International, Inc. taxonomy (NANDA-I) was developed in 1988 and revised in 1996, 2000, 2017 and 2020. In 2023, its title was changed to *Inadequate situational self-esteem* and it currently has a level of evidence of 3.2. It is defined as: “A change from a positive to a negative perception of self-worth, self-acceptance, self-respect, competence and attitude towards oneself in response to a current situation”^(1)^.

Individuals at risk of developing this diagnosis experience personal, spiritual or financial crises and display characteristics such as indecision, excessive obedience, lack of purpose and helplessness^(1)^. In addition, they may show a constant need for approval, difficulty maintaining eye contact^(2)^, insomnia, depressive symptoms, loneliness, mental rumination, verbalizations of self-denial and an inability to deal with adverse situations^(1)^.

University students are a population vulnerable to mental imbalance and crises generated by academic stressors^(3)^. Poor mental health has been associated with a drop in academic performance and withdrawal from studies^(4)^, situations that are particularly relevant in health courses, such as nursing^(5)^.

Although the discussion on mental health in the university population has advanced, there is still a need to deepen knowledge on the subject^(6)^. Although clinical studies show positive results in the management of anxiety, depression and stress^(7-8)^, approaches focused on self-esteem remain scarce. Specifically among undergraduate nursing students, evidence suggests a correlation between suicide risk and low self-esteem^(9)^, highlighting the importance of this construct for student assistance.

This investigation is in line with global research aimed at producing knowledge about nursing diagnoses, improving their components and levels of evidence^(1)^. In addition, it is in line with studies that point to a reduction in self-esteem among nursing students during their undergraduate studies^(9)^.

It is therefore essential to deepen our understanding of situational low self-esteem, identifying predisposing factors and establishing clinical indicators for its early detection in different populations^(10)^. This knowledge can guide the actions of nursing professionals and decision-making for the formulation of qualified care. In the case of students with this diagnosis, the analysis should consider adaptive difficulties, teaching-learning contexts, the predominance of women in nursing courses and social, economic and cultural factors that influence their vulnerability in health^(11-12)^.

Currently, Middle-Range Theories (MRT) have been widely used to validate nursing diagnoses, as they provide a more robust and specific basis for certain populations. They analyze the structure of the diagnoses, the causal factors and their interrelationships, ensuring greater precision and applicability in clinical practice^(10,13)^.

That said, it is believed that the development of an MRT for low self-esteem will improve diagnostic reasoning and contribute to nursing care that is more aligned with the needs of university students. In addition, this theory could support the improvement of the NANDA-I taxonomy and boost the advancement of nursing science.

Thus, this study aims to build a Middle-Range Theory for the nursing diagnosis of low situational self-esteem in nursing students, based on the Roy Adaptation Model (RAM), from the mapping of scientific literature.

## Method

### Study design

This is a methodological study, oriented towards the construction of a MRT, developed from the theoretical-causal model^(13)^ and based on RAM^(14)^.

MRTs are the most robust method for theoretical-causal validation^(1)^, guiding nurses’ actions, strengthening scientific knowledge and improving professional qualification. Its use has become a rigorous method for validating nursing diagnoses, as it aims to reduce the gap between theory and clinical practice by structuring a theoretical gradient^(13)^.

RAM^(14)^ provides the basis for understanding the individual as a system capable of adapting. In this model: a) The person is the recipient of nursing care. b) Health is a state and a continuous process of integration and wholeness. c) The environment encompasses all the conditions and circumstances that influence human behavior and development. d) The goal of nursing is to promote adaptive responses in the four adaptive modes: physiological, self-concept, role function and interdependence^(14)^.

In this sense, the person is seen as a holistic and adaptable system. Roy also pointed out that internal and external stimuli activate regulatory and cognitive mechanisms that work to maintain adaptation. The behavioral responses resulting from this process provide feedback to the individual and the environment, and are categorized either as adaptive responses, which promote integrity, or as ineffective responses, which do not support these goals and therefore require diagnostic nursing action^(10)^.

Based on the identification of these behaviors and reactions to environmental stimuli, nurses determine nursing diagnoses (ND)^(14)^. The ND is a health technology that makes it possible to draw up and implement a care plan, accurately reflecting the patient’s clinical situation. To ensure their effectiveness, the diagnoses need to be validated^(13)^.

In this way, the references adopted are complementary, providing a conceptual framework sufficient to describe, explain and substantiate the diagnosis of situational low self-esteem, establishing a solid link between theory and practice.

In order to build the MRT, the study followed six stages: 1. Definition of the approach for building the theory. 2. Definition of the theoretical-conceptual models. 3. identifying key concepts 4. drawing up a pictorial scheme. 5. formulating propositions and 6. Establishing causal relationships and evidence for practice.

### Data collection, organization and analysis

#### Defining the approach to theory building

This MRT was built on the results of a scoping review. To this end, the research protocol was initially drawn up following the guidelines of the JBI - Manual for Evidence Synthesis^(15)^ and the recommendations of the Preferred Reporting Items for Systematic Reviews and Meta-Analyses extension for Scoping Reviews (PRISMA-ScR): Checklist and Explanation^(16)^. The protocol was registered in the Open Science Framework database, under the identifier DOI 10.17605/OSF.IO/4YRCT.

The research questions were structured based on the PCC mnemonic: P (Population): undergraduate nursing students. C (Concept): situational low self-esteem. C (Context): nursing diagnosis.

With this in mind, the following guiding questions were defined: 1) What are the clinical indicators (consequents), causal factors (antecedents) and essential attributes of the nursing diagnosis of low situational self-esteem in undergraduate nursing students? 2) What are the conceptual and operational definitions of the clinical indicators (consequents) and causal factors (antecedents) of this diagnosis? 3. what are the causal relationships between the diagnosis of low situational self-esteem in undergraduate nursing students?

For the initial search, the PubMed, CINAHL and The Cochrane Library databases were consulted in order to identify the main descriptors and keywords related to the topic. The search strategy was formulated using a combination of Health Sciences Descriptors (DeCS) and Medical Subject Headings (MeSH), resulting in the following search key: Students Nursing OR Student Nursing AND Self Concept OR Self Esteem AND Nursing Diagnosis AND Education Nursing AND Graduation. Adaptations were made according to the database used, with the aim of broadening the scope of the search.

### Inclusion and exclusion criteria

The following were included: a) Publications that addressed the phenomenon of situational low self-esteem in undergraduate nursing students. b) Studies with a quantitative, qualitative or mixed design available entirely in electronic format.

The following were excluded: a) Abstracts, letters to the editor, editorials and opinion pieces. b) Duplicate studies in databases. There was no time or language limit in order to include as many studies as possible.

### Study search and selection procedures

The search for studies was carried out between March 22 and October 24, 2022, with an update between February 3 and 12, 2025, in the following databases: Web of Science, Scopus, PubMed, MEDLINE (Online System for the Search and Analysis of Medical Literature), Science Direct and Google Scholar. The databases were accessed via the CAPES Portal, using the Federated Academic Community (CAFe) platform and the credentials of the Federal University of Rio Grande do Norte.

Initially, the titles and abstracts of the studies identified were evaluated based on the inclusion and exclusion criteria. When there were doubts about eligibility, the study was kept for analysis in the next stage.

The pre-selected studies had their full texts retrieved for full reading and detailed analysis. Duplicates were removed at this stage. The entire process was conducted independently by three researchers.

**Figure 1-:**
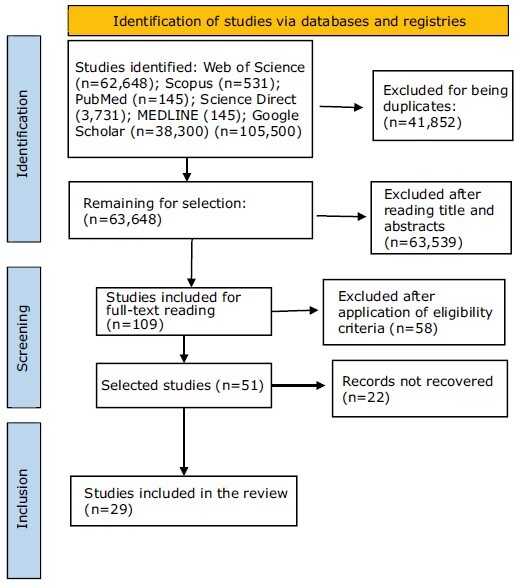
Flowchart of the study selection process according to PRISMA-ScR. Natal, RN, Brazil, 2025

Figure 1 shows the detailed flowchart of the search and sample selection process.

After selecting and including the studies in the review, the main researcher extracted the data, which was recorded in Microsoft Excel spreadsheets. The extraction script included information to characterize the publications (title, authors, year of publication, journal and level of evidence) and to analyze the topic (objective, concepts of self-esteem and low self-esteem). In addition, aspects related to the nursing diagnosis of situational low self-esteem were collected: essential attributes, clinical indicators (consequents), causal factors (antecedents) and their respective definitions.

Based on this analysis, a theoretical gradient was developed, allowing the etiological elements and clinical indicators of the diagnosis of low situational self-esteem to be identified. Causal relationships were also established to explain the occurrence of this diagnosis in nursing students.

### Definition of theoretical-conceptual models

The review made it possible to identify specific elements of low situational self-esteem in undergraduate nursing students. These elements were analyzed on the basis of RAM^(14)^ and the NANDA-I^(1)^ diagnosis of low situational self-esteem, providing the basis for the construction of the MRT.

### Definition of main concepts

After the operational definition of the concepts, the contents were analyzed and integrated to structure the MRT, considering three main components: 1- Antecedents: factors that influence the development of situational low self-esteem; 2- Consequents: behaviors or reactions resulting from this diagnosis. 3- Essential attributes: fundamental characteristics of the phenomenon.

Key concepts related to the topic were selected, categorizing them as stimuli (antecedents) and behaviors (consequents), as well as defining their respective conceptualizations and operationalizations.

### Construction of a pictorial scheme

To make it easier to understand the relationships between the concepts, a pictorial diagram was drawn up illustrating the connections between the attributes, antecedents and consequents identified in the scoping review, allowing for a better visualization of the causal relationships in the public studied.

### Construction of propositions

Propositions are statements that describe relationships between concepts within a theory, allowing for the formulation of empirically testable hypotheses^(17)^. In this stage, explanatory propositions were drawn up about the connections between essential attributes, clinical antecedents and clinical consequents, based on the findings of the scoping review and the author’s interpretation.

### Establishing causal relationships and evidence for practice

The causal relationships between the elements of the theory, which cannot always be fully visualized in the diagram, were described. These relationships were structured using assertive statements, enabling logical and verifiable clinical reasoning^(13)^.

### Ethical aspects

As this is a scoping review based on publicly accessible secondary sources, the study does not need to be approved by the Research Ethics Committee. Nevertheless, the ethical aspects were rigorously respected, especially considering the copyright of the studies, with due citation and referencing of the scientific productions used.

## Results

The Middle-Range Theory for the nursing diagnosis of low situational self-esteem among nursing students presented five attributes, 15 antecedents and 23 clinical consequents. In this sense, a pictogram, eight propositions and 12 causal relationships and evidence for practice were developed, from the perspective of RAM, in a connection between theory and practice. This articulation contributes to translating knowledge into relevant actions for undergraduate nursing students, in a more holistic way.

### Defining the approach to theory building

The MRT for the nursing diagnosis of low situational self-esteem among nursing students is prescriptive and has a deductive orientation, as it was built from the results of the literature mapping.

A sample of 29 studies was obtained. The highest concentration of publications was from 2016 to 2020 (44.8%). A geographical distribution was identified with the highest frequency of studies on the subject on the Asian continent (41.3%), followed by Europe (31.0%; n=9).

As for the language, 79.3% of the articles were in English. The most frequent research method was cross-sectional, with 48.2%, reflecting the evidence classification level 4b^(17)^. The studies involved nursing students from different undergraduate courses. The Rosenberg Self-Esteem Scale was the most widely used instrument for assessing self-esteem.

### Definition of theoretical-conceptual models and key concepts

The essential attributes identified were: negative feelings (48.3%); negative evaluations of self-worth (27.6%); pessimistic perceptions (17.2%); impaired psychosocial well-being (10.3%); and level of self-esteem (6.9%). Thus, these attributes were grouped together by the main researcher and the definition of the diagnosis under study was formed: “Level of self-esteem expressed by negative feelings, pessimistic perceptions and negative evaluations of self-worth, which reflects on impaired psychosocial well-being”.

Next, 15 antecedents were identified, also known as causal factors. These were subdivided and organized according to RAM, called stimuli and classified as focal, contextual and residual, as shown in Table 1.

**Table 1-:** Antecedents of the concept of low situational self-esteem identified in the scoping review. Natal, RN, Brazil, 2025

Antecedents		n*	%
Focal stimuli			
1	Depression	10	34.4
2	Anxiety	4	13.8
3	Individual in psychological distress	4	13.8
4	Personal dissatisfaction	4	13.8
5	Introspective personality	2	6.9
Contextual stimuli			
6	Socio-economic vulnerability	7	24.1
7	Fragile relationships	7	24.1
8	Traumatic experiences	7	24.1
9	Stressful environments	7	24.1
10	Low perception of family support	5	17.2
11	Excessive use of smartphones	2	6.9
12	Insufficient social support	2	6.9
13	Receiving and verbalizing negative words	2	6.9
14	Bullying	2	6.9
Residual stimuli			
15	Imbalance in the role	2	6.9

*n=29

As a result, 23 consequents were found, also known as clinical indicators. These were organized as behaviors according to RAM’s adaptive modes, as shown in Table 2.

**Table 2-:** Consequences of low situational self-esteem identified in the scoping review. Natal, RN, Brazil, 2025

Consequences		n*	%
Behaviors - physiological mode			
1	Poor sleep quality	2	6.9
2	Eating disorders	1	3.45
Behaviors - self-concept mode			
3	Non-assertive behavior	13	43.3
4	Negative emotions	11	36.6
5	Depressive symptoms	10	34.4
6	Stress	8	27.6
7	Anxious symptoms	4	13.8
8	Suicidal ideation	4	13.8
9	Emotional instability	4	13.8
10	Low empathy	3	10.3
11	Catastrophism	3	10.3
12	Failures in interpersonal communication	3	10.3
13	Underestimates ability to cope	3	10.3
14	Change in body image	2	6.9
15	Low self-confidence	2	6.9
16	Low satisfaction	2	6.9
17	Fear of negative evaluation	2	6.9
Behaviors - role performance mode			
18	Poor performance	10	34.4
19	Poor adaptation	7	24.1
20	Evasion	3	10.3
21	Ineffective coping	3	10.3
22	Deficit in self-care	2	6.9
Behaviors - interdependence mode			
23	Difficulties in establishing bonds	4	13.8

*n=29

**Figure 2-:**
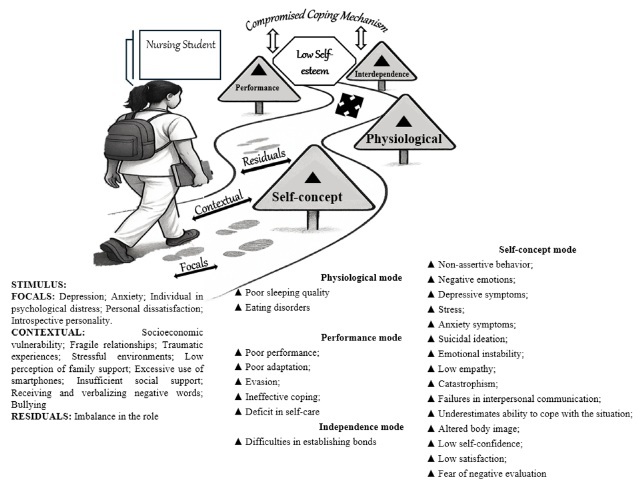
Pictogram of situational low self-esteem in adolescents. Natal, RN, Brazil, 2025

*MRT = Middle-Range Theory

### Pictorial scheme (pictogram)

In order to explain the relationship between the key concepts of MRT (attributes, antecedents and consequents) and the concepts of RAM (stimuli and behaviors), the pictogram shown in Figure 2 was constructed.

The pictogram represents the nursing student’s journey, carrying with it their personal experiences. During this journey, the student is exposed to stimuli that activate coping mechanisms. However, when these mechanisms are compromised, they can lead to the development of situational low self-esteem, affecting adaptive modes - self-concept, role performance, physiological and interdependence - and triggering isolated or combined reactions.

In the proposed Middle-Range Theory, the elements of Roy’s Adaptation Model^(14)^ are applied as follows:

- Person: Represented by the undergraduate nursing student with low situational self-esteem, considered a holistic and adaptive system.

- Environment: Includes both the internal world (the student’s perceptions, emotions and beliefs) and the external world (the academic, social and cultural context).

- Health: Refers to the student’s ability to adapt to constant changes in the internal and external environments.

- Nursing goal: To promote adaptive responses that help the student balance adaptive modes, favoring adaptation and well-being.

### Formulating propositions

The propositions formulated in this MRT were:

Nursing students receive stimuli resulting from their immersion in life contexts (personal, family, social, professional), which can cause low situational self-esteem, resulting in non-assertive behaviors.

Low situational self-esteem can occur due to the action of one or more stimuli/background (focal, contextual, residual), producing one or more behaviors/consequences.

If the student is in psychological distress, has lived through traumatic experiences; has received and/or verbalized negative words and has had an imbalance in their role, then they are more susceptible to developing situational low self-esteem.

Nursing students have personal aspects which, together with the contextual ones, enhance negative self-concept and imbalance in health care, resulting in depression, stress, anxiety and even suicidal ideation.

Nursing students with low situational self-esteem may show instability in their role performance by displaying non-assertive behaviors, communication failures, poor performance and even dropping out of school.

Nursing students with low situational self-esteem have personal factors, which together with exposure to social determinants of health, influence the physiological mode, causing poor sleep quality, eating disorders and biopsychosocial imbalance.

Students with low situational self-esteem, because they perceive the relationships and judgments of others as negative for them, find it difficult to establish bonds.

The nurse should contribute to the student’s coping balance, promoting the integration of self-esteem.

### Causal relationships and evidence for practice

In order to expand the theoretical basis of the MRT, causal relationships were established between the etiological factors and clinical indicators of situational low self-esteem in nursing students, as shown in Figure 3.

**Figure 3-:** Causal relationships established between etiological factors and clinical indicators of situational low self-esteem. Natal, RN, Brazil, 2025

Causal relationships
1. Students in psychological distress are more likely to develop situational low self-esteem, which can lead to poor performance and maladaptation in the academic context ^( 18 - 20 )^ . These students also have a biopsychosocial imbalance and can develop anxiety, depression and suicidal ideation ^( 21 )^ .
2. The role of the student can be unbalanced when experiencing stressful environments and suffering practices such as bullying. These circumstances project situational low self-esteem onto the individual, which can lead to poor sleep quality, eating disorders, anxious and depressive symptoms, stress, changes in body image, fear of negative evaluation, poor performance and academic dropout ^( 20 - 22 )^ .
3. Traumatic experiences can have a significant impact on a student’s self-esteem. This can trigger situational low self-esteem, which can lead to the expression of negative emotions, emotional instability, low self-confidence, low satisfaction, low empathy and suicidal ideation ^( 19 , 23 - 24 )^ .
4. Students with an introspective personality are susceptible to low situational self-esteem, and may fail in interpersonal communication, catastrophize and underestimate their ability to deal with the situation ^( 24 - 26 )^ .
5. Nursing students who receive and often verbalize negative words are susceptible to situational low self-esteem, which can lead to emotional instability, negative emotions, anxious symptoms and interpersonal communication failures ^( 22 )^ .
6. The excessive use of smartphones makes nursing students susceptible to situational low self-esteem and increases the chances of depression and suicidal ideation ^( 25 )^ .
7. Socioeconomic vulnerability conditions situations in students’ lives, which leave them susceptible to situational low self-esteem, leading to behaviors such as catastrophism and poor sleep quality ^( 25 , 27 )^ .
8. Students with personal dissatisfaction tend to have low self-esteem, altered body image perception and eating disorders ^( 27 )^ .
9. The student with low situational self-esteem who is personally dissatisfied somatizes attitudes of low self-confidence, underestimates the ability to cope with the situation, ineffective coping and deficits in self-care ^( 25 , 28 )^ .
10. Academics with low self-esteem who have a history of fragile relationships tend to have difficulties in establishing bonds ^( 22 , 25 )^ .
11. Nursing students with low situational self-esteem, who experience a low perception of family support and insufficient social support, tend to exhibit behaviors such as: stress; non-assertive behaviors; poor adaptation; low self-confidence; low academic performance; ineffective coping and dropout ^( 20 - 22 , 28 )^ .
12. Nursing students predisposed to mental illness can trigger situational low self-esteem. This can be driven by living in stressful environments, anxiety, depression and/or bullying; resulting in anxious, depressive symptoms and stress ^( 22 , 24 - 25 , 28 )^ .

Thus, for the diagnosis of situational low self-esteem in undergraduate nursing students, the construction of the MRT made it possible to study its main causes and consequences, so that nurses have adequate tools with levels of evidence to favor health care for nursing students.

## Discussion

The MRT of the diagnosis of interest was made up of five attributes, 15 antecedents and 23 clinical consequents, a pictogram, eight propositions and 12 causal relationships and evidence for practice, from the perspective of Roy’s Adaptation Model, in a connection between theory and practice. This articulation contributes to translating knowledge into relevant actions for undergraduate nursing students, in a more holistic way, as well as advancing knowledge in nursing.

Self-esteem is an indicator that reflects mental health, as it measures psychological stability in the face of various challenging aspects of life^(29)^. Among the etiological factors (antecedents) identified in this study, the following prevailed: stressful learning environments, ineffective experiences, problematic relationships, socioeconomic vulnerability and low perception of family support.

Stressful learning environments can be understood as educational settings in teaching institutions and professional practices in health services, which favor the appearance of stress in nursing students^(30)^. This factor is relevant because both the different environments and contexts and the involvement in processes and results that affect individuals (such as emotions and feelings) have repercussions on students’ low self-esteem^(31)^.

University students may react heterogeneously to academic experiences: some will adapt quickly and efficiently; others will travel this path slowly; others will not adapt completely, leading to dropouts^(32)^. Lack of financial resources and expectations in relation to training are factors that can make it difficult and discourage students from prioritizing completing the course^(33)^. Interpersonal relationships can be a determining factor in the decision to drop out of university, as well as maladaptation to teaching strategies^(34)^.

University students’ lifestyles can be influenced by the university environment and the experiences they have had there, with the adoption of healthy or unhealthy practices^(35)^. These experiences are influenced by a transitory emotional state. It is emphasized that high levels of negative affect indicate a greater frequency and intensity of unpleasant experiences and displeasure, and people in this condition consider themselves sad, discouraged and worried^(36)^.

Students with low situational self-esteem tend to have interference in the quality of their relationships^(37)^. Nursing students with low self-esteem have significant impacts on social outcomes, worsening when associated with low social support and family support^(38)^. Perceived social support can act as a buffer for situational low self-esteem and other mental health conditions, as its positive perception provides a sense of belonging and strengthens the mental health of university students, enabling them to cope with the usual demands of everyday life^(39)^.

With regard to the mental health of university students, it has become an increasingly recurrent issue around the world, and has been identified as a major public health problem. One study identified a high prevalence of psychological distress in first-year health science students compared to students from other areas^(40)^. Psychological distress among university students is related to various factors, such as academic pressure, coping skills, economics and social communication. In turn, those susceptible to mental problems are more likely to have negative self-assessments^(41)^.

As for the indicators resulting from the diagnosis of low situational self-esteem in nursing, this study showed relational characteristics relating to poor adaptation to academic life and psychosocial aspects of nursing undergraduates, highlighting the prevalence of: non-assertive behaviors, negative emotions, depression, low academic performance, stress and poor adaptation to university.

According to the authors^(42)^, self-esteem is a psychological phenomenon with a close relationship between the emotional and cognitive dimensions, and every time there is negative reinforcement of personal actions, there is a greater propensity for self-esteem to decline. Therefore, people with low situational self-esteem are dissatisfied with themselves, have emotional instability, impaired performance in their personal, family, social, school and professional lives, understanding of themselves and others, acceptance of self-criticism and tolerance of problems^(43)^.

One investigation revealed that all participants with excessive internet use had a significantly higher frequency of symptoms of depression, anxiety and stress^(44)^. The results of a literature review also showed that bullying is an important trigger of low self-esteem in nursing in their academic contexts^(45)^.

In addition, this nursing diagnosis can identify personal characteristics such as: negative self-assessment; indecisive behaviour; non-assertive behaviour; verbalization of feelings of worthlessness; excessive search for reaffirmation; excessive conformism; dependence on the opinion of others; passivity; verbalization of feelings of guilt; verbalization of feelings of shame^(46)^.

The interface of low self-esteem with other nursing diagnoses was mentioned in another study, such as anxiety; ineffective communication; ineffective coping; depression, decreased involvement in recreational activities; stress, social isolation; ineffective relationships^(1)^. It is important to identify the relationships between conditions associated with the nursing diagnosis of low self-esteem, facilitating the definition of interventions aimed at the needs of clients^(10)^.

One study classified factors that affected the self-esteem of nursing students into two groups: one positive, called protection, and the other negative, called pressure. Among the pressure factors were: low student self-efficacy, a sense of triviality, ineffective interaction between teacher and student and low self-confidence. Protective factors included the acquisition of knowledge, professional autonomy, religious beliefs and interest in choosing nursing^(9)^.

A study of 569 Peruvian university students showed changes when comparing variables such as emotional exhaustion and self-esteem in relation to academic satisfaction^(47)^. Another study of undergraduate nursing students in Pakistan^(48)^ found that individuals with low self-esteem showed levels of suicidal behavior associated with depression and anxiety. Among the main factors that led to these findings are excessive workload, close contact with teachers, colleagues and patients, insecurity and fear at the time of patient care, low self-esteem and other feelings.

Therefore, the academic situations experienced by nursing students have the potential to develop low self-esteem, which has an impact on stress and poor sleep quality, as well as their negative physical and emotional health outcomes^(26)^.

Understanding the relationships between the antecedents and consequents of the diagnosis was essential for building the theoretical predictive structure of the causal relationships established in these components. Low situational self-esteem in nursing students can contribute to nurses’ diagnostic inference in student health care.

The study’s limitations include: the extraction of key concepts (attributes, antecedents and consequents) only by the main researcher, without the help of other researchers, the topic being little explored, as well as the small amount of previous research on the diagnosis of low situational self-esteem, which demonstrates that this study fills a gap and serves as a basis for the emergence of new investigations to deepen the theme.

The MRT constructed in this study could contribute to nursing practice by making it possible to identify the factors, among those presented by the student, which probably caused the diagnosis. This information could help to draw up an individualized and efficient care plan focusing on etiological factors that are sensitive to nursing interventions.

Furthermore, the pictogram symbolizes the causal relationships established between the antecedents and consequents of the diagnosis. This representation helps to effectively verify the distribution of clinical indicators in the dimensions of the diagnosis, as well as the causal hierarchy of etiological factors.

## Conclusion

The mapping of scientific productions in the context of nursing diagnoses of low self-esteem in nursing students showed little exploration of the subject, both nationally and internationally.

The MRT developed for the nursing diagnosis of low situational self-esteem in nursing students presented 5 attributes, 15 antecedents and 23 clinical consequents. In addition, an illustrative pictogram, 8 propositions and 12 causal relationships and evidence for practice were developed, based on RAM, promoting the connection between theory and practice. This approach makes it possible to translate scientific knowledge into concrete actions, enabling more holistic and targeted care for undergraduate nursing students.

The construction of the MRT enabled an in-depth understanding of the main causes and consequences of the diagnosis of situational low self-esteem in nursing students. As a result, nurses now have validated, evidence-based tools to improve health care for this population and strengthen nursing science.

Furthermore, we encourage the development of future studies aimed at the empirical validation of this diagnosis, ensuring its applicability and effectiveness in clinical practice.
