# Supplementary material for: Middle-range theory of the diagnosis of low situational self-esteem in undergraduate nursing students
Source: Rev Lat Am Enfermagem. 2025 Aug 18;33:e4650. doi: 10.1590/1518-8345.7662.4650 (PMC12364456; doi:10.1590/1518-8345.7662.4650)
Supplement: Translation

# Translation: Teoria de Médio Alcance do diagnóstico de baixa autoestima situacional nos estudantes de graduação em enfermagem*

**DOI:** 10.1590/1518-8345.7662.4651

**Authors:** Laís Vasconcelos Santos, Layane Ryane da Silva Souza, Marcos Manoel Sousa Silva, Marcos Venícios de Oliveira Lopes, Ana Luisa Brandão de Carvalho Lira

**Affiliations:** 1 Universidade Federal do Rio Grande do Norte, Natal, RN, Brasil.; 2 Universidade Federal do Ceará, Fortaleza, CE, Brasil.; 3 Bolsista do Conselho Nacional de Desenvolvimento Científico e Tecnológico (CNPq), Brasil.

**Keywords:** Teoria de Enfermagem, Autoimagem, Estudantes de Enfermagem, Diagnóstico de Enfermagem, Pesquisa em Enfermagem, Processo de Enfermagem

## Abstract

construir uma Teoria de Médio Alcance para o diagnóstico de baixa autoestima situacional em estudantes de enfermagem, a partir do mapeamento da literatura científica.

estudo metodológico voltado à construção de uma Teoria de Médio Alcance, desenvolvido com base no modelo teórico-causal e fundamentado no Modelo de Adaptação de Roy. O processo foi operacionalizado em seis etapas: (1) definição da abordagem para construção da teoria; (2) definição dos modelos teórico-conceituais; (3) identificação dos conceitos-chave; (4) elaboração de um esquema pictórico; (5) formulação de proposições; e (6) estabelecimento de relações causais e evidências para a prática.

foram identificados cinco atributos essenciais, 15 antecedentes e 23 consequentes clínicos. A análise desses elementos possibilitou o desenvolvimento da Teoria de Médio Alcance, composta por um diagrama ilustrativo, oito proposições, 12 relações causais e evidências para a prática.

o pequeno número de estudos mostra a pouca exploração do tema no âmbito nacional e internacional. A Teoria de Médio Alcance ampliou a compreensão do diagnóstico de baixa autoestima situacional, oferecendo subsídios para a atuação do enfermeiro no contexto universitário.

## Destaques

**(1)** A TMA introduz elementos para o enfermeiro utilizar na prática clínica. **(2)** Amplia a compreensão sobre o diagnóstico baixa autoestima situacional. **(3)** Apoia o avanço da prática e da pesquisa relacionada ao processo de cuidar estudantil. **(4)** Pode servir como recurso orientador para implementar ações frente a baixa autoestima.

## Introdução

O diagnóstico de enfermagem *Baixa autoestima situacional* da taxonomia da NANDA International, Inc. (NANDA-I) foi desenvolvido em 1988 e revisado nos anos de 1996, 2000, 2017 e 2020. Em 2023, seu título foi alterado para *Autoestima inadequada situacional* e, atualmente, possui nível de evidência 3.2. É definido como: “Mudança de percepção positiva para negativa sobre o valor próprio, a autoaceitação, o autorrespeito, a competência e a atitude em relação a si mesmo, em resposta a uma situação atual”^(1)^.

Indivíduos em risco de desenvolver esse diagnóstico vivenciam crises pessoais, espirituais ou financeiras e apresentam características como indecisão, obediência excessiva, falta de propósito e desamparo^(1)^. Além disso, podem apresentar necessidade constante de aprovação, dificuldade em manter contato visual^(2)^, insônia, sintomas depressivos, solidão, ruminação mental, verbalizações de autonegação e incapacidade de lidar com situações adversas^(1)^.

Os estudantes universitários constituem uma população vulnerável ao desequilíbrio mental e às crises geradas pelos estressores acadêmicos^(3)^. A má saúde mental tem sido associada à queda no desempenho acadêmico e ao abandono dos estudos^(4)^, situações especialmente relevantes em cursos da área da saúde, como a enfermagem^(5)^.

Embora a discussão sobre saúde mental na população universitária tenha avançado, ainda há necessidade de aprofundar o conhecimento sobre o tema^(6)^. Ainda que os estudos clínicos mostrem resultados positivos no gerenciamento da ansiedade, depressão e estresse^(7-8)^, as abordagens focadas na autoestima permanecem escassas. Especificamente entre graduandos de enfermagem, evidências sugerem uma correlação entre risco de suicídio e baixa autoestima^(9)^, ressaltando a importância desse constructo para a assistência estudantil.

A investigação se alinha a pesquisas globais voltadas à produção de conhecimento sobre diagnósticos de enfermagem, aprimorando seus componentes e níveis de evidência^(1)^. Além disso, ela converge com estudos que apontam a redução da autoestima entre estudantes de enfermagem ao longo da graduação^(9)^.

Portanto, torna-se essencial aprofundar a compreensão da baixa autoestima situacional, identificando fatores predisponentes e estabelecendo indicadores clínicos para sua detecção precoce em diferentes populações^(10)^. Tal conhecimento pode orientar as ações dos profissionais de enfermagem e a tomada de decisões para a formulação de cuidados qualificados. No caso dos estudantes com esse diagnóstico, a análise deve considerar dificuldades adaptativas, contextos de ensino-aprendizagem, predominância feminina nos cursos de enfermagem e fatores sociais, econômicos e culturais que influenciam sua vulnerabilidade em saúde^(11-12)^.

Atualmente, as Teorias de Médio Alcance (TMA) têm sido amplamente utilizadas na validação de diagnósticos de enfermagem, pois possibilitam uma fundamentação mais robusta e específica para determinadas populações. Elas analisam a estrutura dos diagnósticos, os fatores causais e suas inter-relações, garantindo maior precisão e aplicabilidade na prática clínica^(10,13)^.

Dito isso, acredita-se que o desenvolvimento de uma TMA da baixa autoestima aprimorará o raciocínio diagnóstico e contribuirá para uma assistência de enfermagem mais alinhada às necessidades dos universitários. Além disso, essa teoria poderá subsidiar o aprimoramento da taxonomia da NANDA-I e impulsionar o avanço da ciência da enfermagem.

Assim, este estudo tem como objetivo construir uma Teoria de Médio Alcance para o diagnóstico de enfermagem baixa autoestima situacional em estudantes de enfermagem, fundamentada no Modelo de Adaptação de Roy (MAR), a partir do mapeamento da literatura científica.

## Método

### Desenho do estudo

Trata-se de um estudo metodológico, orientado para a construção de uma TMA, desenvolvido a partir do modelo teórico-causal^(13)^ e fundamentado no MAR^(14)^.

As TMA são o método mais robusto para validação teórico-causal^(1)^, orientando as ações do enfermeiro, fortalecendo o conhecimento científico e aprimorando a qualificação profissional. Seu uso tornou-se um método rigoroso na validação de diagnósticos de enfermagem, pois visa reduzir a lacuna entre teoria e prática clínica, estruturando um gradiente teórico^(13)^.

O MAR^(14)^ fornece a base para compreender o indivíduo como um sistema capaz de se adaptar. Nesse modelo: a) A pessoa é a receptora dos cuidados de enfermagem. b) A saúde é um estado e um processo contínuo de integração e totalidade. c) O ambiente abrange todas as condições e circunstâncias que influenciam o comportamento e o desenvolvimento humano. d) A meta da enfermagem é promover respostas adaptativas nos quatro modos adaptativos: fisiológico, autoconceito, função de papel e interdependência^(14)^.

Nesse sentindo, a pessoa é vista como um sistema holístico e adaptável. Roy ainda abordou que os estímulos internos e externos ativam mecanismos reguladores e cognitivos que atuam para manter a adaptação. As respostas comportamentais resultantes desse processo fornecem retroalimentação ao indivíduo e ao ambiente, sendo categorizadas ou como respostas adaptativas, que promovem a integridade, ou como respostas inefetivas, que não sustentam essas metas e, portanto, exigem ação diagnóstica da enfermagem^(10)^.

Com base na identificação desses comportamentos e reações aos estímulos ambientais, o enfermeiro determina os diagnósticos de enfermagem (DE)^(14)^. O DE é uma tecnologia em saúde que possibilita a elaboração e implementação de um plano de cuidados, refletindo com precisão a situação clínica do paciente. Para garantir sua eficácia, os diagnósticos precisam ser validados^(13)^.

Dessa forma, os referenciais adotados são complementares, fornecendo estrutura conceitual suficiente para descrever, explicar e fundamentar o diagnóstico de baixa autoestima situacional, estabelecendo um vínculo sólido entre teoria e prática.

Para a construção da TMA, o estudo seguiu seis etapas: 1. Definição da abordagem para construção da teoria. 2. Definição dos modelos teórico-conceituais. 3. Identificação de conceitos-chave. 4. Elaboração de um esquema pictórico. 5. Formulação de proposições e 6. Estabelecimento de relações causais e evidências para a prática.

### Coleta, organização e análise dos dados

#### Definição da abordagem para a construção da teoria

A presente TMA foi construída a partir dos resultados de uma revisão de escopo. Para isso, elaborou-se inicialmente o protocolo de pesquisa, seguindo as diretrizes do *JBI - Manual for Evidence Synthesis* ^(15)^ e as recomendações do *Preferred Reporting Items for Systematic Reviews and Meta-Analyses extension for Scoping Reviews (PRISMA-ScR): Checklist and Explanation* ^(16)^. O protocolo foi registrado na base de dados *Open Science Framework*, sob o identificador DOI 10.17605/OSF.IO/4YRCT.

As questões de pesquisa foram estruturadas com base no mnemônico PCC: P (População): estudantes de graduação em enfermagem. C (Conceito): baixa autoestima situacional. C (Contexto): diagnóstico de enfermagem.

Com isso, definiram-se as seguintes perguntas norteadoras: 1. Quais são os indicadores clínicos (consequentes), os fatores causais (antecedentes) e os atributos essenciais do diagnóstico de enfermagem de baixa autoestima situacional em estudantes de graduação em enfermagem? 2. Quais são as definições conceituais e operacionais dos indicadores clínicos (consequentes) e dos fatores causais (antecedentes) desse diagnóstico? 3. Quais são as relações causais do diagnóstico baixa autoestima situacional em estudantes de graduação em enfermagem?

Para a busca inicial, consultaram-se as bases de dados *PubMed*, CINAHL e *The Cochrane Library*, a fim de identificar os principais descritores e palavras-chave relacionadas ao tema. A estratégia de busca foi formulada a partir da combinação dos Descritores em Ciências da Saúde (DeCS) e dos *Medical Subject Headings* (MeSH), resultando na seguinte chave de pesquisa: *Students Nursing OR Student Nursing AND Self Concept OR Self Esteem AND Nursing Diagnosis AND Education Nursing AND Graduation.* Adaptações foram realizadas conforme a base de dados utilizada, com o objetivo de ampliar a abrangência da pesquisa.

### Critérios de inclusão e exclusão

Foram incluídos: a) Publicações que abordassem o fenômeno baixa autoestima situacional em estudantes de graduação em enfermagem. b) Estudos com delineamento quantitativo, qualitativo ou mistos disponíveis integralmente em formato eletrônico.

Foram excluídos: a) Os tipos: resumos, cartas ao editor, editoriais e artigos de opinião. b) Os estudos duplicados em bases de dados. Não houve limite temporal ou de idioma, visando a inclusão do maior número possível de trabalhos.

### Procedimentos de busca e seleção dos estudos

A busca pelos estudos foi realizada entre 22 de março a 24 de outubro de 2022, com uma atualização entre 03 a 12 de fevereiro de 2025, nas seguintes bases de dados: *Web of Science*, *Scopus*, *PubMed*, MEDLINE (Sistema Online de Busca e Análise de Literatura Médica), *Science Direct* e *Google Scholar*. O acesso às bases foi realizado via Portal CAPES, utilizando a plataforma Comunidade Acadêmica Federada (CAFe) e credenciais da Universidade Federal do Rio Grande do Norte.

Inicialmente, os títulos e resumos dos estudos identificados foram avaliados com base nos critérios de inclusão e exclusão. Quando havia dúvidas quanto à elegibilidade, o estudo era mantido para análise na etapa subsequente. Os estudos pré-selecionados tiveram seus textos completos recuperados para leitura integral e análise detalhada. A remoção de duplicatas foi realizada nesta fase. Todo o processo foi conduzido por três pesquisadores de forma independente.

A Figura 1 apresenta o fluxograma detalhado do processo de busca e seleção da amostra.

**Figura 1-:** Fluxograma do processo de seleção dos estudos de acordo com o PRISMA-ScR. Natal, RN, Brasil, 2025

Após a seleção e inclusão dos estudos na revisão, a pesquisadora principal realizou a extração dos dados, os quais foram registrados em planilhas no *Microsoft Excel*. O roteiro de extração incluiu informações para a caracterização das publicações (título, autores, ano de publicação, periódico e nível de evidência) e para a análise do tema (objetivo, conceitos de autoestima e baixa autoestima). Além disso, coletou-se aspectos relacionados ao diagnóstico de enfermagem baixa autoestima situacional: atributos essenciais, indicadores clínicos (consequentes), fatores causais (antecedentes) e suas respectivas definições.

A partir dessa análise, foi desenvolvido um gradiente teórico, permitindo a identificação dos elementos etiológicos e dos indicadores clínicos do diagnóstico baixa autoestima situacional. Ainda, estabeleceu-se relações causais que explicam a ocorrência desse diagnóstico em estudantes de enfermagem.

### Definição dos modelos teórico-conceituais

A revisão permitiu identificar elementos específicos da baixa autoestima situacional em estudantes de enfermagem durante a graduação. Esses elementos foram analisados com base no MAR^(14)^ e no diagnóstico baixa autoestima situacional da NANDA-I^(1)^, fornecendo a base para a construção da TMA.

### Definição dos principais conceitos

Após a definição operacional dos conceitos, os conteúdos foram analisados e integrados para estruturar a TMA, considerando três componentes principais: 1- Antecedentes: fatores que influenciam o desenvolvimento da baixa autoestima situacional; 2- Consequentes: comportamentos ou reações decorrentes desse diagnóstico. 3- Atributos essenciais: características fundamentais do fenômeno.

Foram selecionados conceitos-chave relacionados ao tema, categorizando-os como estímulos (antecedentes) e comportamentos (consequentes), além de definir suas respectivas conceituações e operacionalizações.

### Construção de um esquema pictorial

Para facilitar a compreensão das relações entre os conceitos, foi elaborado um diagrama pictórico que ilustra as conexões entre atributos, antecedentes e consequentes identificados na revisão de escopo, permitindo uma melhor visualização das relações causais no público estudado.

### Construção de proposições

As proposições são afirmações que descrevem relações entre conceitos dentro de uma teoria, permitindo a formulação de hipóteses testáveis empiricamente^(17)^. Na referida etapa, foram elaboradas proposições explicativas sobre as conexões entre atributos essenciais, antecedentes e consequentes clínicos, com base nos achados da revisão de escopo e na interpretação da autoria.

### Estabelecimento das relações de causalidade e evidências para a prática

Foram descritas as relações de causalidade entre os elementos da teoria, que, nem sempre, podem ser completamente visualizadas no diagrama. Essas relações foram estruturadas por meio de declarações assertivas, possibilitando um raciocínio clínico lógico e verificável^(13)^.

### Aspectos éticos

Por se tratar de uma revisão de escopo baseada em fontes secundárias de acesso público, o estudo dispensa aprovação pelo Comitê de Ética em Pesquisa. Não obstante, os aspectos éticos foram rigorosamente respeitados, sobretudo considerando os direitos autorais dos estudos, com a devida citação e referenciamento das produções científicas utilizadas.

## Resultados

A Teoria de Médio Alcance para o diagnóstico de enfermagem baixa autoestima situacional de estudantes de enfermagem apresentou cinco atributos, 15 antecedentes e 23 consequentes clínicos. Nesse sentido, foi desenvolvido um pictograma, oito proposições e 12 relações causais e evidências para a prática, sob a ótica do MAR, em uma conexão entre teoria e prática. Essa articulação contribui na tradução do conhecimento em ações pertinentes para os graduandos em enfermagem, de maneira mais holística.

### Definição da abordagem para a construção de teoria

A TMA do diagnóstico de enfermagem baixa autoestima situacional de estudantes de enfermagem é prescritiva e conjugou a orientação dedutiva, pois foi construída a partir dos resultados do mapeamento da literatura.

Assim, obteve-se uma amostra de 29 estudos. A maior concentração de publicações foi no período de 2016 a 2020 (44,8%). Identificou-se uma distribuição geográfica com maior frequência de estudos sobre o assunto no continente asiático (41,3%), seguido do europeu (31,0%; n=9).

Quanto ao idioma, 79,3% dos artigos estavam em inglês. O método mais frequente das pesquisas foi o transversal, com 48,2%, refletindo-se na classificação de evidência nível 4b^(17)^. As pesquisas envolveram estudantes de enfermagem em diferentes períodos dos cursos de graduação. A escala de Autoestima de Rosenberg foi o instrumento para avaliação da autoestima mais adotado.

### Definição dos modelos teórico-conceituais e conceitos-chave

Os atributos essenciais identificados foram: sentimentos negativos (48,3%); avaliações negativas do valor de si (27,6%); percepções pessimistas (17,2%); bem-estar psicossocial prejudicado (10,3%); e, nível de autoestima (6,9%). Assim, esses atributos foram agrupados pela pesquisadora principal e constitui-se a definição do diagnóstico em estudo: “Nível da autoestima expresso por sentimentos negativos, percepções pessimistas e avaliações negativas do valor de si, que reflete no bem-estar psicossocial prejudicado”.

Na sequência, foram identificados 15 antecedentes, também denominados de fatores causais. Estes foram subdivididos e organizados conforme o MAR, nomeando-os de estímulo e classificando-os em focal, contextual e residual, conforme Tabela 1.

**Tabela 1-:** Antecedentes do conceito baixa autoestima situacional identificados na revisão de escopo. Natal, RN, Brasil, 2025

Antecedentes		n*	%
Estímulos focais			
1	Depressão	10	34,4
2	Ansiedade	4	13,8
3	Individuo em sofrimento psíquico	4	13,8
4	Insatisfação pessoal	4	13,8
5	Personalidade introspectiva	2	6,9
Estímulos contextuais			
6	Vulnerabilidade socioeconômica	7	24,1
7	Relacionamentos fragilizados	7	24,1
8	Experiências traumáticas	7	24,1
9	Ambientes estressantes	7	24,1
10	Baixa percepção de apoio familiar	5	17,2
11	Uso excessivo de *smartphones*	2	6,9
12	Apoio social insuficiente	2	6,9
13	Receber e verbalizar palavras negativas	2	6,9
14	*Bullying*	2	6,9
Estímulos residuais			
15	Desequilíbrio no papel	2	6,9

*n=29

Por conseguinte, verificou-se a presença de 23 consequentes, também denominados indicadores clínicos. Esses foram organizados como comportamentos conforme os modos adaptativos do MAR, como apresentado na Tabela 2.

**Tabela 2-:** Consequentes da baixa autoestima situacional identificados na revisão de escopo. Natal, RN, Brasil, 2025

Consequentes		n*	%
Comportamentos – modo fisiológico			
1	Má qualidade do sono	2	6,9
2	Distúrbios alimentares	1	3,45
Comportamentos – modo autoconceito			
3	Comportamentos não assertivos	13	43,3
4	Emoções negativas	11	36,6
5	Sintomas depressivos	10	34,4
6	Estresse	8	27,6
7	Sintomas ansiosos	4	13,8
8	Ideação suicida	4	13,8
9	Instabilidade emocional	4	13,8
10	Baixa empatia	3	10,3
11	Catastrofismo	3	10,3
12	Falhas na comunicação interpessoal	3	10,3
13	Subestima a capacidade de lidar com a situação	3	10,3
14	Alteração na imagem corporal	2	6,9
15	Baixa autoconfiança	2	6,9
16	Baixa satisfação	2	6,9
17	Medo de avaliação negativa	2	6,9
Comportamentos – modo desempenho de papel			
18	Baixo desempenho	10	34,4
19	Má adaptação	7	24,1
20	Evasão	3	10,3
21	Enfrentamento ineficaz	3	10,3
22	Déficit no autocuidado	2	6,9
Comportamentos – modo interdependência			
23	Dificuldades em estabelecer vínculos	4	13,8

*n=29

### Esquema pictorial (pictograma)

Para explanar a relação entre os conceitos-chave da TMA (atributos, antecedentes e consequentes) com os conceitos do MAR (estímulos e comportamentos), construiu-se o pictograma apresentado na Figura 2.

O pictograma construído representa a jornada do estudante de enfermagem, carregando consigo as suas experiências pessoais. Durante essa caminhada, o aluno é exposto a estímulos que ativam mecanismos de enfrentamento. No entanto, quando esses mecanismos são comprometidos, eles podem levar ao desenvolvimento da baixa autoestima situacional, afetando os modos adaptativos — autoconceito, desempenho de papel, fisiológico e interdependência — e desencadeando reações isoladas ou combinadas.

Na Teoria de Médio Alcance proposta, os elementos do Modelo de Adaptação de Roy^(14)^ são aplicados da seguinte forma:

- Pessoa: Representada pelo estudante de graduação em enfermagem com baixa autoestima situacional, considerado um sistema holístico e adaptativo.
- Ambiente: Inclui tanto o mundo interno (percepções, emoções e crenças do estudante) quanto o mundo externo (contexto acadêmico, social e cultural).
- Saúde: Refere-se à capacidade de adaptação do estudante frente às constantes mudanças nos ambientes interno e externo.
- Meta da enfermagem: Promover respostas adaptativas que auxiliem o estudante a equilibrar os modos adaptativos, favorecendo a adaptação e o bem-estar.

**Figura 2-:** Pictograma da TMA* baixa autoestima situacional em adolescentes. Natal, RN, Brasil, 2025

*TMA = Teoria de Médio Alcance

### Formulação de proposições

As proposições formuladas na TMA foram:

1. O estudante de enfermagem recebe estímulos resultantes da sua imersão nos contextos de vida (pessoal, familiar, social, profissional), os quais podem proporcionar a baixa autoestima situacional, resultando em comportamentos não assertivos.
2. A baixa autoestima situacional pode ocorrer por ação de um ou mais estímulos/antecedente (focais, contextuais, residuais), produzindo um ou mais comportamentos/consequentes.
3. Se o estudante está em sofrimento psíquico, vivenciou experiências traumáticas; recebeu e/ou verbalizou palavras negativas e teve desequilíbrio no seu papel, então está mais suscetível a desenvolver a baixa autoestima situacional.
4. O estudante de enfermagem apresenta aspectos pessoais, que em conjunto aos contextuais, potencializam o autoconceito negativo e o desequilíbrio na atenção à saúde, resultando em depressão, estresse, ansiedade e até ideação suicida.
5. O estudante de enfermagem com baixa autoestima situacional poderá apresentar instabilidade no seu desempenho de papel ao apresentar comportamentos não assertivos, falhas na comunicação, baixo desempenho e até evasão dos estudos.
6. O estudante de enfermagem com baixa autoestima situacional possui fatores pessoais, que em conjunto a exposição de determinantes sociais de saúde, influenciam o modo fisiológico, causando má qualidade do sono, distúrbios alimentares e desequilíbrio biopsicossocial.
7. O estudante com baixa autoestima situacional, por compreender as relações e julgamentos dos outros como negativas para si, apresenta dificuldades em estabelecer vínculos.
8. O enfermeiro deverá contribuir com o equilíbrio do enfrentamento do estudante, promovendo a integração da autoestima.

### Relações causais e evidências para a prática

Com vistas à ampliação da fundamentação teórica da TMA, estabeleceram-se as relações causais entre os fatores etiológicos e indicadores clínicos da baixa autoestima situacional em estudantes de enfermagem, conforme apresenta a Figura 3.

**Figura 3-:** Relações causais estabelecidas entre os fatores etiológicos e indicadores clínicos da baixa autoestima situacional. Natal, RN, Brasil, 2025

Relações causais	
1. O estudante em sofrimento psíquico está mais propenso a desenvolver a baixa autoestima situacional, o que pode levar ao baixo desempenho e a má adaptação no contexto acadêmico ^( 18 - 20 )^ . Esse aluno também apresenta desequilíbrio biopsicossocial, podendo desenvolver ansiedade, depressão e ideação suicida ^( 21 )^ .	
2. O papel do estudante pode ser desequilibrado ao vivenciar ambientes estressantes e sofrer práticas como o *bullying.* Essas circunstâncias projetam no indivíduo a baixa autoestima situacional, podendo levá-lo a apresentar: má qualidade no sono; distúrbios alimentares, sintomas ansiosos e depressivos, estresse, alterações na imagem corporal, medo de avaliação negativa, baixo desempenho e evasão acadêmica ^( 20 - 22 )^ .	
3. As experiências traumáticas podem ter um impacto significativo na autoestima do estudante. Isso pode despertar a baixa autoestima situacional, podendo levar a expressão de emoções negativas, instabilidade emocional, baixa autoconfiança, baixa satisfação, baixa empatia e ideação suicida ^( 19 , 23 - 24 )^ .	
4. O estudante de personalidade introspectiva é suscetível à baixa autoestima situacional, podendo apresentar falhas na comunicação interpessoal, catastrofismo e subestimar a capacidade de lidar com a situação ^( 24 - 26 )^ .	
5. O estudante de enfermagem que recebe e costuma verbalizar palavras negativas está passível à baixa autoestima situacional, o que pode impulsionar a instabilidade emocional, as emoções negativas, os sintomas ansiosos e as falhas na comunicação interpessoal ^( 22 )^ .	
6. O uso excessivo de *smartphones* torna o estudante de enfermagem suscetível à baixa autoestima situacional e aumenta as chances do risco de depressão e ideação suicida ^( 25 )^ .	
7. A vulnerabilidade socioeconômica condiciona situações na vida estudantil, as quais deixam-na passível à baixa autoestima situacional proporcionando comportamentos como o catastrofismo e a má qualidade do sono do estudante ^( 25 , 27 )^ .	
8. O estudante que apresenta insatisfação pessoal tende à baixa autoestima, com alterações na percepção da imagem corporal e distúrbios alimentares ^( 27 )^ .	
9. O aluno com baixa autoestima situacional que está com insatisfação pessoal, somatiza atitudes de baixa autoconfiança, subestima a capacidade de lidar com a situação, enfrentamento ineficaz e déficit no autocuidado ^( 25 , 28 )^ .	
10. O acadêmico com baixa autoestima que possui histórico de relacionamentos fragilizados, tende a apresentar dificuldades em estabelecer vínculos ^( 22 , 25 )^ .	
11. O estudante de enfermagem com baixa autoestima situacional que vivencia a baixa percepção de apoio familiar e o apoio social insuficiente, tem tendência a apresentar comportamentos como: estresse; comportamentos não assertivos; má adaptação; baixa autoconfiança; baixo desempenho acadêmico; enfrentamento ineficaz e evasão ^( 20 - 22 , 28 )^ .	
12. O estudante de enfermagem predisposto ao adoecimento mental pode desencadear a baixa autoestima situacional. Esta pode ser impulsionada pela vivência em ambientes estressantes, pela ansiedade, pela depressão e/ou *bullying;* resultando em sintomas ansiosos, depressivos e estresse ^( 22 , 24 - 25 , 28 )^ .	

Dessa maneira, para o diagnóstico de baixa autoestima situacional em estudantes de graduação em Enfermagem, a construção da TMA possibilitou o estudo das suas principais causas e consequências, para que o enfermeiro possua ferramentas adequadas e com níveis de evidência para favorecer a assistência à saúde aos estudantes de enfermagem.

## Discussão

A TMA do diagnóstico de interesse foi composta por cinco atributos, 15 antecedentes e 23 consequentes clínicos, um pictograma, oito proposições e 12 relações causais e evidências para a prática, sob a ótica do Modelo de Adaptação de Roy, em uma conexão entre teoria e prática. Essa articulação contribui na tradução do conhecimento em ações pertinentes para os graduandos em enfermagem, de maneira mais holística, bem como para o avanço do conhecimento na Enfermagem.

A autoestima é um indicador que reflete a saúde mental, pois mede a estabilidade psíquica diante de vários aspectos desafiadores da vida^(29)^. Dentre os fatores etiológicos (antecedentes) identificados nesta pesquisa, prevaleceram: ambientes de aprendizagem estressantes, as experiências ineficazes, os relacionamentos problemáticos, vulnerabilidade socioeconômica e baixa percepção de apoio familiar.

Os ambientes de aprendizagem estressantes podem ser entendidos como os cenários educacionais nas instituições de ensino e práticas profissionais nos serviços de saúde, as quais favorecem o aparecimento do estresse nos estudantes de enfermagem^(30)^. Tal fator tem relevância, pois tanto os diferentes ambientes e contextos quanto o envolvimento em processos e resultados que afetam os indivíduos (como emoções e sentimentos) repercutem na baixa autoestima dos estudantes^(31)^.

Os universitários podem vir a reagir de maneira heterogênea às experiências acadêmicas: alguns vão adaptar-se rápida e eficientemente; outros vão percorrer esse caminho lentamente; outros não se adaptarão completamente, ocasionando evasões^(32)^. A falta de recursos financeiros e as expectativas em relação à formação são fatores que podem dificultar e desestimular o discente a priorizar a conclusão do curso^(33)^. Relacionamentos interpessoais podem ser determinantes para a decisão de abandono do curso universitário, bem como inadaptação às estratégias de ensino^(34)^.

O estilo de vida do estudante universitário pode ser influenciado pelo ambiente universitário e as experiências vividas nele, com adoção de práticas saudáveis ou prejudiciais à saúde^(35)^. Essas experiências são influenciadas por um estado emocional transitório. Enfatiza-se que níveis elevados de afetos negativos indicam a maior frequência e intensidade de experiências desagradáveis e de desprazer, e pessoas nessa condição consideram-se tristes, desanimadas e preocupadas^(36)^.

Os alunos com baixa autoestima situacional tendem a possuir interferências na qualidade dos relacionamentos^(37)^. Os estudantes de enfermagem com baixa autoestima apresentam repercussões significativas nos resultados sociais, tendo piora quando relacionado ao baixo apoio social e suporte familiar^(38)^. O apoio social percebido pode funcionar como amortecedor para baixa autoestima situacional e outras condições de saúde mental, pois sua percepção positiva proporciona um senso de pertencimento e fortalece a saúde mental dos estudantes universitários, permitindo-lhes lidar com as exigências habituais da vida cotidiana^(39)^.

Em relação à saúde mental dos estudantes universitários, nota-se que é um tema cada vez mais recorrente no mundo, sendo apontado como um importante problema de saúde pública. Um estudo identificou alta prevalência de sofrimento psicológico em estudantes no primeiro ano de faculdade ciências da saúde comparado aos alunos de outras áreas^(40)^. O sofrimento psíquico entre estudantes universitários está relacionado a vários fatores, como: pressão acadêmica, habilidades de enfrentamento, economia e comunicação social. Por sua vez, os sujeitos suscetíveis a problemas mentais apresentam maior probabilidade de auto avaliações negativas^(41)^.

Quanto aos indicadores consequentes do diagnóstico de baixa autoestima situacional em Enfermagem, o presente estudo mostrou características relacionais referentes à má adaptação de vida acadêmica e a aspectos psicossociais dos graduandos em enfermagem, destacando-se a prevalência de: comportamentos não assertivos, emoções negativas, depressão, baixo desempenho acadêmico, estresse e má adaptação à universidade.

Para autores^(42)^, a autoestima corresponde a um fenômeno psicológico com íntima relação entre as dimensões emocionais e cognitivas, cada vez que existe reforço negativo face às ações pessoais, maior propensão para o declínio da autoestima. Logo, pessoas com baixa autoestima situacional apresentam-se insatisfeitos consigo, possuem instabilidade emocional, prejuízo no desempenho da sua vida pessoal, familiar, social, escolar, profissional, na compreensão de si e dos outros, na aceitação de autocrítica e tolerância aos problemas^(43)^.

Uma investigação revelou que todos os participantes com uso excessivo de *internet* apresentaram uma frequência significativamente maior de sintomas de depressão, ansiedade e estresse^(44)^. Os resultados de uma revisão de literatura também sinalizaram que o *bullying* se configura como um importante desencadeador da baixa autoestima na enfermagem em seus contextos acadêmicos^(45)^.

Ademais, poderão ser identificados, nesse diagnóstico de enfermagem, características pessoais como: avaliação negativa de si; comportamento indeciso; comportamento não assertivo; verbalização de sentimentos de inutilidade, procura excessiva de reafirmação; excessivo conformismo; dependência da opinião de terceiros; passividade; verbalização de sentimento de culpa; verbalização de sentimentos de vergonha^(46)^.

A interface da baixa autoestima com outros diagnósticos de enfermagem foi referida em outro estudo tais como a ansiedade; comunicação ineficaz; *coping* não eficaz; depressão, envolvimento em atividades de recreação diminuída; estresse, isolamento social; relacionamento ineficaz^(1)^. Ressalta-se a importância da identificação das relações de condições associadas ao diagnósticos de enfermagem da baixa autoestima, facilitando a definição de intervenções direcionadas às necessidades dos clientes^(10)^.

Um estudo classificou fatores que afetaram a autoestima de estudantes de enfermagem em dois grupos, a saber: um positivo chamado de proteção, e outro negativo chamado de pressão. Dentre os fatores de pressão foram destacados: baixa autoeficácia dos alunos, senso de trivialidade, interação ineficaz entre professor e aluno e baixa autoconfiança. Já os fatores de proteção incluíram aquisição de conhecimento, autonomia profissional, crenças religiosas e o interesse ao escolher a área de enfermagem^(9)^.

Um estudo com 569 universitários peruanos mostrou alterações quando foram comparadas as variáveis como esgotamento emocional e autoestima relacionados à satisfação acadêmica^(47)^. Uma outra investigação realizada com estudantes de graduação em enfermagem no Paquistão^(48)^ apresentou que há, em indivíduos com baixa autoestima, a presença de níveis de comportamento suicida associados à depressão e ansiedade. Dentre os principais fatores que levaram a esses achados estão o excesso de carga horária, contato íntimo com professores, colegas e pacientes, insegurança e medo no momento do atendimento do paciente, baixa autoestima e outros sentimentos.

Portanto, as situações acadêmicas vivenciadas pelos estudantes de enfermagem apresentam potencial de desenvolvimento para a baixa autoestima que possuem impacto no estresse e a má qualidade do sono, bem como seus desfechos negativos à saúde física e emocional^(26)^.

A compreensão das relações entre antecedentes e consequentes do diagnóstico foi essencial para construção da estrutura teórica preditiva das relações causais estabelecidas nesses componentes. A TMA baixa autoestima situacional em estudantes de enfermagem pode contribuir na inferência diagnóstica dos enfermeiros na assistência à saúde estudantil.

Como limitações do estudo, destacam-se: a extração dos conceitos-chave (atributos, antecedentes e consequentes) apenas pelo pesquisador principal, sem o auxílio de outros pesquisadores, o tema ser pouco explorado, bem como pequeno número de pesquisas prévias sobre o diagnóstico baixa autoestima situacional, o que mostra que este estudo vem preencher uma lacuna e servir de fundamentação para o surgimento de novas investigações para aprofundamento temático.

A TMA construída neste estudo poderá contribuir para a prática do enfermeiro, possibilitando a identificação dos fatores, dentre os apresentados pelo estudante, que provavelmente causaram o diagnóstico. Essa informação poderá fornecer subsídios para a elaboração de um plano de cuidados individualizado e eficiente com foco em fatores etiológicos sensíveis às intervenções de enfermagem.

Outrossim, o pictograma simboliza as relações de causalidade estabelecidas entre os antecedentes e os consequentes do diagnóstico. Essa representação auxilia na verificação eficaz da distribuição dos indicadores clínicos nas dimensões do diagnóstico, assim como a hierarquia causal dos fatores etiológicos.

## Conclusão

O mapeamento das produções científicas no contexto dos diagnósticos de enfermagem sobre a baixa autoestima em estudantes de enfermagem mostrou pouca exploração do tema, tanto em âmbito nacional quanto internacionalmente.

A TMA desenvolvida para o diagnóstico de enfermagem baixa autoestima situacional em estudantes de enfermagem apresentou 5 atributos, 15 antecedentes e 23 consequentes clínicos. Além disso, elaborou-se um pictograma ilustrativo, 8 proposições e 12 relações causais e evidências para a prática, fundamentadas no MAR, promovendo a conexão entre teoria e prática. Tal abordagem permite traduzir o conhecimento científico em ações concretas, possibilitando uma assistência mais holística e direcionada aos graduandos em enfermagem.

A construção da TMA possibilitou uma compreensão aprofundada das principais causas e consequências do diagnóstico de baixa autoestima situacional em estudantes de enfermagem. Dessa forma, os enfermeiros passam a dispor de ferramentas validadas e baseadas em evidências para qualificar a assistência à saúde desse público e fortalecer a Ciência da Enfermagem.

Ademais, incentiva-se o desenvolvimento de estudos futuros voltados à validação empírica deste diagnóstico, garantindo sua aplicabilidade e eficácia na prática clínica.

# Translation: Teoría de alcance medio del diagnóstico baja autoestima situacional en estudiantes de pregrado en enfermería*

**DOI:** 10.1590/1518-8345.7662.4649

**Authors:** Laís Vasconcelos Santos, Layane Ryane da Silva Souza, Marcos Manoel Sousa Silva, Marcos Venícios de Oliveira Lopes, Ana Luisa Brandão de Carvalho Lira

**Affiliations:** 1 Universidade Federal do Rio Grande do Norte, Natal, RN, Brasil.; 2 Universidade Federal do Ceará, Fortaleza, CE, Brasil.; 3 Becaria (o) del Conselho Nacional de Desenvolvimento Científico e Tecnológico (CNPq), Brasil.

**Keywords:** Teoría de Enfermería, Autoimagen, Estudiantes de Enfermería, Diagnóstico de enfermería, Investigación en Enfermería, Proceso de Enfermería

## Abstract

construir una teoría de alcance medio para el diagnóstico de enfermería baja autoestima situacional en estudiantes de enfermería a partir del mapeo de la literatura científica.

estudio metodológico orientado a la construcción de una teoría de alcance medio, desarrollado con base en el modelo teórico-causal y fundamentado en el Modelo de Adaptación de Roy. El proceso se operacionalizó en seis etapas: (1) definición del abordaje para la construcción de la teoría; (2) definición de los modelos teórico-conceptuales; (3) identificación de los conceptos clave; (4) elaboración de un esquema pictórico; (5) formulación de proposiciones; y (6) establecimiento de relaciones causales y evidencias para la práctica.

se identificaron cinco atributos esenciales, 15 antecedentes y 23 consecuentes clínicos. El análisis de estos elementos posibilitó el desarrollo de la teoría de alcance medio, compuesta por un diagrama ilustrativo, ocho proposiciones, 12 relaciones causales y evidencias para la práctica.

el reducido número de estudios demuestra la escasa exploración del tema en las realidades nacional e internacional. La teoría de alcance medio amplió la comprensión del diagnóstico baja autoestima situacional, ofreciendo subsidios para la actuación del enfermero en el contexto universitario.

## Destacados

**(1)** La TMA introduce elementos para que el enfermero los utilice en la práctica clínica. **(2)** Amplía la comprensión sobre el diagnóstico baja autoestima situacional. **(3)** Apoya el avance de la práctica y de la investigación relacionada con el proceso de cuidado estudiantil. **(4)** Puede servir como recurso orientador para implementar acciones ante la baja autoestima.

## Introducción

El diagnóstico de enfermería *Baja autoestima situacional* de la taxonomía de NANDA International, Inc. (NANDA-I) fue desarrollado en 1988 y revisado en los años 1996, 2000, 2017 y 2020. En 2023, su título se cambió a *Autoestima inadecuada situacional* y actualmente posee nivel de evidencia 3.2. Se define como “Cambio de la percepción positiva a negativa acerca del propio valor, la autoaceptación, el autorrespeto, la competencia y la actitud hacia uno mismo en respuesta a una situación actual”^(1)^.

Los individuos en riesgo de desarrollar este diagnóstico experimentan crisis personales, espirituales o financieras y presentan características como indecisión, obediencia excesiva, falta de propósito y desamparo^(1)^. Además, pueden mostrar necesidad constante de aprobación, dificultad para mantener contacto visual^(2)^, insomnio, síntomas depresivos, soledad, rumiación mental, verbalizaciones de autonegación e incapacidad para afrontar situaciones adversas^(1)^.

Los estudiantes universitarios constituyen una población vulnerable al desequilibrio mental y a las crisis provocadas por estresores académicos^(3)^. La mala salud mental se ha asociado con la disminución del rendimiento académico y el abandono de los estudios^(4)^, situaciones especialmente relevantes en carreras del área de la salud como enfermería^(5)^.

Aunque la discusión sobre salud mental en la población universitaria ha avanzado, aún es necesario profundizar el conocimiento sobre este tema^(6)^. Estudios clínicos muestran resultados positivos en el manejo de la ansiedad, la depresión y el estrés^(7-8)^, pero las estrategias centradas en la autoestima siguen siendo escasas. Específicamente entre estudiantes de enfermería, la evidencia sugiere una correlación entre riesgo de suicidio y baja autoestima^(9)^, lo que resalta la importancia de este constructo para la atención estudiantil.

Esta investigación se alinea con estudios globales destinados a producir conocimiento sobre diagnósticos de enfermería, perfeccionando sus componentes y niveles de evidencia^(1)^. Además, converge con trabajos que indican la reducción de la autoestima entre estudiantes de enfermería a lo largo de la carrera^(9)^.

Por lo tanto, resulta esencial profundizar la comprensión de la baja autoestima situacional, identificar factores predisponentes y establecer indicadores clínicos para su detección precoz en diferentes poblaciones^(10)^. Este conocimiento puede orientar las acciones de los profesionales de enfermería y la toma de decisiones para formular cuidados de calidad. En el caso de los estudiantes con este diagnóstico, el análisis debe considerar dificultades adaptativas, contextos de enseñanza-aprendizaje, predominio femenino en los cursos de enfermería y factores sociales, económicos y culturales que influyen en su vulnerabilidad en salud^(11-12)^.

Actualmente, las Teorías de Alcance Medio (TAM) se utilizan ampliamente en la validación de diagnósticos de enfermería, pues permiten una fundamentación más sólida y específica para poblaciones concretas. Analizan la estructura de los diagnósticos, los factores causales y sus interrelaciones, garantizando mayor precisión y aplicabilidad en la práctica clínica^(10,13)^.

Dicho esto, se considera que el desarrollo de una TAM sobre baja autoestima situacional mejorará el razonamiento diagnóstico y contribuirá a una asistencia de enfermería más alineada con las necesidades de los universitarios. Además, esta teoría podrá respaldar el perfeccionamiento de la taxonomía de NANDA-I e impulsar el avance de la ciencia de la enfermería.

Así, este estudio tiene como objetivo construir una Teoría de Alcance Medio para el diagnóstico de enfermería baja autoestima situacional en estudiantes de enfermería, fundamentada en el Modelo de Adaptación de Roy (MAR), a partir del mapeo de la literatura científica.

## Método

### Diseño del estudio

Se trata de un estudio metodológico orientado a la construcción de una TAM, desarrollado a partir del modelo teórico-causal^(13)^ y fundamentado en el MAR^(14)^.

Las TAM son el método más robusto para la validación teórico-causal^(1)^, orientando las acciones del enfermero, fortaleciendo el conocimiento científico y mejorando la cualificación profesional. Su uso se ha convertido en un método riguroso en la validación de diagnósticos de enfermería, pues pretende reducir la brecha entre la teoría y la práctica clínica, estructurando un gradiente teórico^(13)^.

El MAR^(14)^ proporciona la base para comprender al individuo como un sistema capaz de adaptarse. En este modelo: a) la persona es la receptora de los cuidados de enfermería; b) la salud es un estado y un proceso continuo de integración y totalidad; c) el ambiente abarca todas las condiciones y circunstancias que influyen en el comportamiento y el desarrollo humano; d) la meta de la enfermería es promover respuestas adaptativas en los cuatro modos adaptativos: fisiológico, autoconcepto, función de rol e interdependencia^(14)^.

En este sentido, la persona es vista como un sistema holístico y adaptable. Roy también señaló que los estímulos internos y externos activan mecanismos reguladores y cognitivos que actúan para mantener la adaptación. Las respuestas conductuales resultantes de este proceso proporcionan retroalimentación al individuo y al ambiente, siendo categorizadas como respuestas adaptativas, que promueven la integridad, o respuestas inefectivas, que no sostienen esas metas y, por lo tanto, requieren acción diagnóstica de enfermería^(10)^.

Con base en la identificación de estos comportamientos y reacciones a los estímulos ambientales, el enfermero determina los diagnósticos de enfermería (DE)^(14)^. El DE es una tecnología en salud que posibilita la elaboración e implementación de un plan de cuidados, reflejando con precisión la situación clínica del paciente. Para garantizar su eficacia, estos diagnósticos necesitan ser validados^(13)^.

De este modo, los marcos de referencia adoptados son complementarios, proporcionando una estructura conceptual suficiente para describir, explicar y fundamentar el diagnóstico de baja autoestima situacional, estableciendo un vínculo sólido entre teoría y práctica.

Para la construcción de la TAM, el estudio siguió seis etapas: 1. Definición del abordaje para la construcción de la teoría. 2. Definición de los modelos teórico-conceptuales. 3. Identificación de conceptos clave. 4. Elaboración de un esquema pictórico. 5. Formulación de proposiciones. 6. Establecimiento de relaciones causales y evidencias para la práctica.

### Recolección, organización y análisis de los datos

#### Definición del abordaje para la construcción de la teoría

La presente TAM se construyó a partir de los resultados de una revisión de alcance. Para ello, se elaboró inicialmente el protocolo de investigación siguiendo las directrices del *JBI Manual for Evidence Synthesis* ^(15)^ y las recomendaciones del *Preferred Reporting Items for Systematic Reviews and Meta-Analyses extension for Scoping Reviews* (PRISMA-ScR): *Checklist and Explanation* ^(16)^. Este protocolo se registró en la base de datos *Open Science Framework*, bajo el identificador DOI 10.17605/OSF.IO/4YRCT.

Las preguntas de investigación se estructuraron con base en el mnemónico PCC: P (Población): estudiantes de pregrado en enfermería. C (Concepto): baja autoestima situacional. C (Contexto): diagnóstico de enfermería.

Con ello se definieron las siguientes preguntas orientadoras: 1. ¿Cuáles son los indicadores clínicos (consecuentes), los factores causales (antecedentes) y los atributos esenciales del diagnóstico de enfermería baja autoestima situacional en estudiantes de pregrado en enfermería? 2. ¿Cuáles son las definiciones conceptuales y operativas de los indicadores clínicos (consecuentes) y de los factores causales (antecedentes) de este diagnóstico? 3. ¿Cuáles son las relaciones causales del diagnóstico baja autoestima situacional en estudiantes de pregrado en enfermería?

Para la búsqueda inicial se consultaron las bases de datos PubMed, CINAHL y The Cochrane Library con el fin de identificar los principales descriptores y palabras clave relacionadas con el tema. La estrategia de búsqueda se formuló a partir de la combinación de los Descriptores en Ciencias de la Salud (DeCS) y los *Medical Subject Headings* (MeSH), resultando en la siguiente ecuación de búsqueda: *Students Nursing OR Student NursingANDSelf ConceptORSelf EsteemANDNursing DiagnosisANDEducation NursingANDGraduation*. Se realizaron adaptaciones conforme a la base de datos utilizada con el objetivo de ampliar el alcance de la investigación.

### Criterios de inclusión y exclusión

Se incluyeron: a) Publicaciones que abordaran el fenómeno de la baja autoestima situacional en estudiantes de pregrado en enfermería. b) Estudios con diseño cuantitativo, cualitativo o mixto disponibles íntegramente en formato electrónico.

Se excluyeron: a) Resúmenes, cartas al editor, editoriales y artículos de opinión. b) Estudios duplicados en bases de datos. No hubo límite temporal ni de idioma, con el fin de incluir el mayor número posible de trabajos.

### Procedimientos de búsqueda y selección de los estudios

La búsqueda de los estudios se realizó del 22 de marzo al 24 de octubre de 2022, con una actualización del 03 al 12 de febrero de 2025, en las siguientes bases de datos: *Web of Science*, *Scopus*, *PubMed*, MEDLINE (Sistema en Línea de Búsqueda y Análisis de Literatura Médica), *Science Direct* y *Google Scholar*. El acceso a las bases se llevó a cabo mediante el Portal CAPES, utilizando la plataforma *Comunidade Acadêmica Federada* (CAFe) y credenciales de la *Universidade Federal do Rio Grande do Norte*.

Inicialmente, los títulos y resúmenes de los estudios identificados se evaluaron según los criterios de inclusión y exclusión. Cuando existían dudas sobre la elegibilidad, el estudio se mantenía para su análisis en la etapa siguiente.

Los estudios preseleccionados tuvieron sus textos completos recuperados para lectura integral y análisis detallado. La eliminación de duplicados se realizó en esta fase. Todo este proceso fue llevado a cabo por tres investigadores de forma independiente.

La Figura 1 presenta el diagrama de flujo detallado del proceso de búsqueda y selección de la muestra.

**Figura 1-:** Diagrama de flujo del proceso de selección de los estudios de acuerdo con el PRISMA-ScR. Natal, RN, Brasil, 2025

Tras la selección e inclusión de los estudios en la revisión, la investigadora principal realizó la extracción de datos, los cuales se registraron en hojas de cálculo de Microsoft Excel. El guion de extracción incluyó información para la caracterización de las publicaciones (título, autores, año de publicación, revista y nivel de evidencia) y para el análisis del tema (objetivo, conceptos de autoestima y baja autoestima). Además, se recopilaron aspectos relacionados con el diagnóstico de enfermería baja autoestima situacional: atributos esenciales, indicadores clínicos (consecuentes), factores causales (antecedentes) y sus respectivas definiciones.

A partir de este análisis se desarrolló un gradiente teórico que permitió identificar los elementos etiológicos y los indicadores clínicos del diagnóstico baja autoestima situacional. Asimismo, se establecieron relaciones causales que explican la ocurrencia de este diagnóstico en estudiantes de enfermería.

### Definición de los modelos teórico-conceptuales

La revisión permitió identificar elementos específicos de la baja autoestima situacional en estudiantes de enfermería durante la graduación. Estos elementos fueron analizados a partir del MAR^(14)^ y del diagnóstico baja autoestima situacional de la NANDA-I^(1)^, proporcionando la base para la construcción de la TAM.

### Definición de los principales conceptos

Tras la definición operacional de los conceptos, los contenidos fueron analizados e integrados para estructurar la TAM, considerando tres componentes principales: 1- Antecedentes: factores que influyen en el desarrollo de la baja autoestima situacional; 2- Consecuentes: comportamientos o reacciones derivadas de este diagnóstico; 3- Atributos esenciales: características fundamentales del fenómeno.

Se seleccionaron conceptos clave relacionados con el tema, categorizándolos como estímulos (antecedentes) y comportamientos (consecuentes), además de definir sus respectivas conceptualizaciones y operacionalizaciones.

### Construcción de un esquema pictórico

Para facilitar la comprensión de las relaciones entre los conceptos, se elaboró un diagrama pictórico. Este pictograma ilustra las conexiones entre atributos, antecedentes y consecuentes identificados en la revisión de alcance, permitiendo una mejor visualización de las relaciones causales en el público estudiado.

### Construcción de proposiciones

Las proposiciones son afirmaciones que describen relaciones entre conceptos dentro de una teoría, posibilitando la formulación de hipótesis empíricamente comprobables^(17)^. En esta etapa se elaboraron proposiciones explicativas sobre las conexiones entre atributos esenciales, antecedentes y consecuentes clínicos, basadas en los hallazgos de la revisión de alcance y en la interpretación de la autoría.

### Establecimiento de las relaciones de causalidad y evidencias para la práctica

Se describieron las relaciones de causalidad entre los elementos de la teoría, que no siempre pueden visualizarse completamente en el diagrama. Estas relaciones se estructuraron mediante declaraciones asertivas, posibilitando un razonamiento clínico lógico y verificable^(13)^.

### Aspectos éticos

Al tratarse de una revisión de alcance basada en fuentes secundarias de acceso público, el estudio prescinde de aprobación por el Comité de Ética en Investigación. No obstante, se respetaron rigurosamente los derechos de autor, con la debida citación y referenciación de las producciones científicas utilizadas.

## Resultados

La teoría de alcance medio para el diagnóstico de enfermería baja autoestima situacional en estudiantes de enfermería presentó cinco atributos, quince antecedentes y veintitrés consecuentes clínicos. Se elaboró un pictograma, ocho proposiciones y doce relaciones causales con evidencias para la práctica, desde la óptica del MAR, en una conexión entre teoría y práctica. Esta articulación contribuye a traducir el conocimiento en acciones pertinentes para los graduandos de enfermería de manera más holística.

### Definición del enfoque para la construcción de la teoría

La TAM del diagnóstico de enfermería baja autoestima situacional en estudiantes de enfermería es prescriptiva y combinó la orientación deductiva, pues se construyó a partir de los resultados del mapeo de la literatura.

Así se obtuvo una muestra de veintinueve estudios. La mayor concentración de publicaciones se observó en el periodo de 2016 a 2020 (44,8%). Se identificó una distribución geográfica con mayor frecuencia de investigaciones sobre el tema en el continente asiático (41,3%), seguido del europeo (31,0%; n = 9).

En cuanto al idioma, el 79,3% de los artículos estaba en inglés. El método más frecuente fue el transversal (48,2%), reflejándose en la clasificación de evidencia nivel 4b^(17)^. Las investigaciones involucraron estudiantes de enfermería en distintos períodos de los cursos de grado. La Escala de Autoestima de Rosenberg fue el instrumento de evaluación de la autoestima más utilizado.

### Definición de los modelos teórico-conceptuales y conceptos clave

Los atributos esenciales identificados fueron: sentimientos negativos (48,3%); evaluaciones negativas del valor de sí (27,6%); percepciones pesimistas (17,2 %); bienestar psicosocial deteriorado (10,3%); y nivel de autoestima (6,9%). Estos atributos se agruparon y constituyen la definición del diagnóstico en estudio: nivel de autoestima expresado por sentimientos negativos, percepciones pesimistas y evaluaciones negativas del valor de sí, que se refleja en un bienestar psicosocial deteriorado.

A continuación se identificaron quince antecedentes, denominados factores causales. Estos se subdividieron y organizaron conforme al MAR, clasificándolos como estímulos focales, contextuales y residuales, tal como se muestra en la Tabla 1.

**Tabla 1-:** Antecedentes del concepto autoestima inadecuada situacional identificados en la revisión de alcance. Natal, RN, Brasil, 2025

Antecedentes		n*	%
Estímulos focales			
1	Depresión	10	34,4
2	Ansiedad	4	13,8
3	Individuo en sufrimiento psíquico	4	13,8
4	Insatisfacción personal	4	13,8
5	Personalidad introspectiva	2	6,9
Estímulos contextuales			
6	Vulnerabilidad socioeconómica	7	24,1
7	Relaciones debilitadas	7	24,1
8	Experiencias traumáticas	7	24,1
9	Ambientes estresantes	7	24,1
10	Baja percepción de apoyo familiar	5	17,2
11	Uso excesivo de *smartphones*	2	6,9
12	Apoyo social insuficiente	2	6,9
13	Recibir y verbalizar palabras negativas	2	6,9
14	*Bullying*	2	6,9
Estímulos residuales			
15	Desequilibrio en el rol	2	6,9

*n=29

En consecuencia, se verificó la presencia de veintitrés consecuentes, también denominados indicadores clínicos. Estos se organizaron como comportamientos según los modos adaptativos del MAR, tal como se presenta en la Tabla 2.

**Tabla 2-:** Consecuentes de la autoestima inadecuada situacional identificados en la revisión de alcance. Natal, RN, Brasil, 2025

Consecuentes		n*	%
Comportamientos – modo fisiológico			
1	Mala calidad del sueño	2	6,9
2	Trastornos alimentarios	1	3,45
Comportamientos – modo autoconcepto			
3	Conductas no asertivas	13	43,3
4	Emociones negativas	11	36,6
5	Síntomas depresivos	10	34,4
6	Estrés	8	27,6
7	Síntomas ansiosos	4	13,8
8	Ideación suicida	4	13,8
9	Inestabilidad emocional	4	13,8
10	Baja empatía	3	10,3
11	Catastrofismo	3	10,3
12	Inestabilidad emocional	3	10,3
13	Subestima la capacidad de afrontar la situación	3	10,3
14	Alteración de la imagen corporal	2	6,9
15	Baja autoconfianza	2	6,9
16	Baja satisfacción	2	6,9
17	Miedo a la evaluación negativa	2	6,9
Comportamientos – modo desempeño de rol			
18	Bajo desempeño	10	34,4
19	Mala adaptación	7	24,1
20	Evasión	3	10,3
21	Afrontamiento ineficaz	3	10,3
22	Déficit en el autocuidado	2	6,9
Comportamientos – modo interdependencia			
23	Dificultades para establecer vínculos	4	13,8

*n=29

### Esquema pictórico (pictograma)

Para exponer la relación entre los conceptos clave de la TAM (atributos, antecedentes y consecuentes) y los conceptos del MAR (estímulos y comportamientos), se construyó el pictograma presentado en la Figura 2.

El pictograma construido representa la trayectoria del estudiante de enfermería, quien lleva consigo sus experiencias personales. Durante este recorrido, el alumno se expone a estímulos que activan mecanismos de afrontamiento; sin embargo, cuando dichos mecanismos se ven comprometidos, pueden conducir al desarrollo de la autoestima inadecuada situacional, afectando los modos adaptativos – autoconcepto, desempeño de rol, fisiológico e interdependencia – y desencadenando reacciones aisladas o combinadas.

En la Teoría de Alcance Medio propuesta, los elementos del Modelo de Adaptación de Roy^(14)^ se aplican de la siguiente forma:

- Persona: representada por el estudiante de pregrado en enfermería con autoestima inadecuada situacional, considerado un sistema holístico y adaptable.
- Ambiente: incluye tanto el mundo interno (percepciones, emociones y creencias del estudiante) como el mundo externo (contexto académico, social y cultural).
- Salud: se refiere a la capacidad de adaptación del estudiante ante los cambios constantes en los entornos interno y externo.
- Meta de la enfermería: promover respuestas adaptativas que ayuden al estudiante a equilibrar los modos adaptativos, favoreciendo la adaptación y el bienestar.

**Figura 2 –:** Pictograma de la TAM autoestima inadecuada situacional en adolescentes. Natal, RN, Brasil, 2025

*TMA = Teoría de Alcance Medio

### Formulación de proposiciones

Las proposiciones formuladas en la presente TAM fueron:

1. El estudiante de enfermería recibe estímulos resultantes de su inmersión en los contextos de vida (personal, familiar, social y profesional) que pueden propiciar la autoestima inadecuada situacional, derivando en conductas no asertivas.
2. La autoestima inadecuada situacional puede originarse por la acción de uno o más estímulos/antecedentes (focales, contextuales o residuales), produciendo uno o más comportamientos/consecuentes.
3. Si el estudiante se encuentra en sufrimiento psíquico, ha vivido experiencias traumáticas, ha recibido y/o verbalizado palabras negativas y ha experimentado desequilibrio en su rol, entonces es más susceptible de desarrollar autoestima inadecuada situacional.
4. El estudiante de enfermería posee aspectos personales que, junto con los contextuales, potencian el autoconcepto negativo y el desequilibrio en la atención a la salud, resultando en depresión, estrés, ansiedad e incluso ideación suicida.
5. El estudiante con autoestima inadecuada situacional puede mostrar inestabilidad en su desempeño de rol al presentar conductas no asertivas, fallos en la comunicación, bajo rendimiento e incluso evasión de los estudios.
6. El estudiante con autoestima inadecuada situacional posee factores personales que, combinados con la exposición a los determinantes sociales de la salud, influyen en el modo fisiológico, causando mala calidad del sueño, trastornos alimentarios y desequilibrio biopsicosocial.
7. El estudiante con autoestima inadecuada situacional, al interpretar las relaciones y los juicios ajenos como negativos para sí, presenta dificultades para establecer vínculos.
8. El enfermero debe contribuir al equilibrio del afrontamiento del estudiante, promoviendo la integración de la autoestima.

### Relaciones causales y evidencias para la práctica

Para ampliar la fundamentación teórica de la TAM, se establecieron las relaciones causales entre los factores etiológicos y los indicadores clínicos de la autoestima inadecuada situacional en estudiantes de enfermería, como se muestra en la Figura 3.

**Figura 3 -:** Relaciones causales establecidas entre los factores etiológicos e indicadores clínicos de la autoestima inadecuada situacional. Natal, RN, Brasil, 2025

Relaciones causales
1. El estudiante en sufrimiento psíquico está más propenso a desarrollar la autoestima inadecuada situacional, lo que puede conducir al bajo desempeño y a la mala adaptación en el contexto académico ^( 18 - 20 )^ . Ese alumno también presenta desequilibrio biopsicosocial, pudiendo desarrollar ansiedad, depresión e ideación suicida ^( 21 )^ .
2. El rol del estudiante puede desequilibrarse al experimentar ambientes estresantes y sufrir prácticas como el *bullying* . Estas circunstancias proyectan en el individuo la autoestima inadecuada situacional, lo que puede llevar a mala calidad del sueño, trastornos alimentarios, síntomas ansiosos y depresivos, estrés, alteraciones en la imagen corporal, miedo a la evaluación negativa, bajo desempeño y evasión académica ^( 20 - 22 )^ .
3. Las experiencias traumáticas pueden impactar significativamente la autoestima del estudiante. Ello puede desencadenar la autoestima inadecuada situacional, dando lugar a la expresión de emociones negativas, inestabilidad emocional, baja autoconfianza, baja satisfacción, baja empatía e ideación suicida ^( 19 , 23 - 24 )^ .
4. El estudiante con personalidad introspectiva es susceptible de desarrollar autoestima inadecuada situacional, pudiendo presentar fallos en la comunicación interpersonal, catastrofismo y subestimar la capacidad de afrontar la situación ^( 24 - 26 )^ .
5. El estudiante de enfermería que recibe y suele verbalizar palabras negativas es propenso a la autoestima inadecuada situacional, lo que puede potenciar la inestabilidad emocional, las emociones negativas, los síntomas ansiosos y los fallos en la comunicación interpersonal ^( 22 )^ .
6. El uso excesivo de *smartphones* vuelve al estudiante de enfermería susceptible de autoestima inadecuada situacional y aumenta las probabilidades de riesgo de depresión e ideación suicida ^( 25 )^ .
7. La vulnerabilidad socioeconómica condiciona situaciones en la vida estudiantil que lo dejan propenso a la autoestima inadecuada situacional, generando comportamientos como el catastrofismo y la mala calidad del sueño ^( 25 , 27 )^ .
El estudiante que presenta insatisfacción personal tiende a la autoestima inadecuada, con alteraciones en la percepción de la imagen corporal y trastornos alimentarios ^( 27 )^ .
El alumno con autoestima inadecuada situacional e insatisfacción personal somatiza actitudes de baja autoconfianza, subestima la capacidad de afrontar la situación, muestra afrontamiento ineficaz y tiene déficit en el autocuidado ^( 25 , 28 )^ .
El estudiante con autoestima inadecuada situacional y antecedentes de relaciones debilitadas tiende a presentar dificultades para establecer vínculos ^( 22 , 25 )^ .
El estudiante de enfermería con autoestima inadecuada situacional, que percibe escaso apoyo familiar y apoyo social insuficiente, tiende a presentar comportamientos como estrés, conductas no asertivas, mala adaptación, baja autoconfianza, bajo desempeño académico, afrontamiento ineficaz y evasión ^( 20 - 22 , 28 )^ .
El estudiante de enfermería predispuesto a la enfermedad mental puede desencadenar la autoestima inadecuada situacional, impulsada por la exposición a ambientes estresantes, ansiedad, depresión y/o *bullying* , lo que resulta en síntomas ansiosos, depresivos y estrés ^( 22 , 24 - 25 , 28 )^ .

De este modo, la construcción de la TAM para el diagnóstico de enfermería autoestima inadecuada situacional en estudiantes de pregrado en enfermería posibilitó el estudio de las principales causas y consecuencias de este diagnóstico, proporcionando al enfermero herramientas adecuadas y con niveles de evidencia que favorezcan la atención a la salud de los estudiantes de enfermería.

## Discusión

La TAM del diagnóstico de interés se compuso de cinco atributos, quince antecedentes y veintitrés consecuentes clínicos, un pictograma, ocho proposiciones y doce relaciones causales y evidencias para la práctica, bajo la óptica del Modelo de Adaptación de Roy, en una conexión entre teoría y práctica. Esta articulación contribuye a traducir el conocimiento en acciones pertinentes para los estudiantes de enfermería de manera más holística, así como al avance del saber en Enfermería.

La autoestima es un indicador que refleja la salud mental, ya que mide la estabilidad psíquica frente a diversos aspectos desafiantes de la vida^(29)^. Entre los factores etiológicos (antecedentes) identificados en esta investigación prevalecieron los ambientes de aprendizaje estresantes, las experiencias ineficaces, las relaciones problemáticas, la vulnerabilidad socioeconómica y la baja percepción de apoyo familiar.

Los ambientes de aprendizaje estresantes pueden entenderse como los escenarios educativos en las instituciones de enseñanza y las prácticas profesionales en los servicios de salud, los cuales han favorecido la aparición del estrés en los estudiantes de enfermería^(30)^. Este factor es relevante porque tanto los distintos entornos y contextos como la implicación en procesos y resultados que afectan a los individuos – tales como emociones y sentimientos – repercuten en la autoestima inadecuada de los estudiantes^(31)^.

Los universitarios pueden reaccionar de forma heterogénea a las experiencias académicas: algunos se adaptarán rápida y eficazmente; otros recorrerán ese camino lentamente; otros no se adaptarán completamente, lo que ocasionará evasiones^(32)^. La falta de recursos financieros y las expectativas respecto a la formación son factores que pueden dificultar y desmotivar al discente para priorizar la finalización del curso^(33)^. Las relaciones interpersonales pueden ser determinantes para la decisión de abandonar la carrera universitaria, así como la inadaptación a las estrategias de enseñanza^(34)^.

El estilo de vida del estudiante universitario puede verse influido por el ambiente universitario y las experiencias vividas en él, con adopción de prácticas saludables o perjudiciales para la salud^(35)^. Dichas experiencias están moduladas por un estado emocional transitorio; niveles elevados de afectos negativos indican una mayor frecuencia e intensidad de vivencias desagradables, y las personas en esa condición se perciben tristes, desanimadas y preocupadas^(36)^.

Los alumnos con autoestima inadecuada situacional tienden a sufrir interferencias en la calidad de sus relaciones^(37)^. Los estudiantes de enfermería con baja autoestima presentan impactos significativos en los resultados sociales, que se agravan cuando se asocian con bajo apoyo social y familiar^(38)^. El apoyo social percibido puede actuar como amortiguador de la autoestima inadecuada situacional y otras condiciones de salud mental, ya que su percepción positiva aporta un sentido de pertenencia y fortalece la salud mental de los estudiantes universitarios, permitiéndoles afrontar las exigencias habituales de la vida cotidiana^(39)^.

En cuanto a la salud mental de los universitarios, se observa que se trata de un tema cada vez más recurrente en todo el mundo y constituye un importante problema de salud pública. Un estudio identificó una elevada prevalencia de sufrimiento psicológico en estudiantes de primer año de carreras de ciencias de la salud en comparación con alumnos de otras áreas^(40)^. El sufrimiento psíquico entre estudiantes universitarios está relacionado con diversos factores, como la presión académica, las habilidades de afrontamiento, la situación económica y la comunicación social. A su vez, los sujetos susceptibles a problemas mentales presentan mayor probabilidad de autoevaluaciones negativas^(41)^.

En cuanto a los indicadores consecuentes del diagnóstico de enfermería autoestima inadecuada situacional, el presente estudio mostró características relacionales vinculadas a la mala adaptación a la vida académica y a los aspectos psicosociales de los estudiantes de enfermería, destacándose la prevalencia de conductas no asertivas, emociones negativas, depresión, bajo rendimiento académico, estrés y mala adaptación a la universidad.

Para algunos autores^(42)^ la autoestima corresponde a un fenómeno psicológico con estrecha relación entre las dimensiones emocionales y cognitivas: a mayor refuerzo negativo frente a las acciones personales, mayor propensión al declive de la autoestima. Así, las personas con autoestima inadecuada situacional se muestran insatisfechas consigo mismas, presentan inestabilidad emocional y deterioro en el desempeño de su vida personal, familiar, social, escolar y profesional, así como en la comprensión de sí mismas y de los demás, en la aceptación de la autocrítica y en la tolerancia a los problemas^(43)^.

Una investigación reveló que todos los participantes con uso excesivo de internet presentaron una frecuencia significativamente mayor de síntomas de depresión, ansiedad y estrés^(44)^. Los resultados de una revisión de la literatura también indicaron que el *bullying* se configura como un importante desencadenante de la autoestima inadecuada en el ámbito académico de la enfermería^(45)^.

Además, en este diagnóstico de enfermería pueden identificarse características personales tales como evaluación negativa de sí mismo, comportamiento indeciso, conducta no asertiva, verbalización de sentimientos de inutilidad, búsqueda excesiva de reafirmación, conformismo excesivo, dependencia de la opinión de terceros, pasividad, verbalización de sentimientos de culpa y de vergüenza^(46)^.

La interfaz de la autoestima inadecuada con otros diagnósticos de enfermería fue mencionada en otro estudio, entre ellos ansiedad, *coping* ineficaz, afrontamiento ineficaz, depresión, disminución de la participación en actividades recreativas, estrés, aislamiento social y relaciones ineficaces^(1)^. Se resalta la importancia de identificar las condiciones asociadas al diagnóstico de autoestima inadecuada para facilitar intervenciones dirigidas a las necesidades de los clientes^(10)^.

Un estudio clasificó los factores que afectan la autoestima de los estudiantes de enfermería en dos grupos: uno positivo, denominado protección, y otro negativo, denominado presión. Entre los factores de presión se destacaron la baja autoeficacia de los alumnos, el sentido de trivialidad, la interacción ineficaz entre profesor y alumno y la baja autoconfianza. Los factores de protección incluyeron la adquisición de conocimientos, la autonomía profesional, las creencias religiosas y el interés al elegir la carrera de enfermería^(9)^.

Un trabajo con 569 universitarios peruanos mostró alteraciones al comparar variables como agotamiento emocional y autoestima relacionadas con la satisfacción académica^(47)^. Otra investigación realizada con estudiantes de enfermería de grado en Pakistán^(48)^ evidenció que los individuos con baja autoestima presentan niveles de conducta suicida asociados a depresión y ansiedad. Entre los principales factores que condujeron a estos hallazgos figuran la sobrecarga horaria, el contacto estrecho con profesores, compañeros y pacientes, la inseguridad y el miedo durante la atención al paciente, la baja autoestima y otros sentimientos.

Por tanto, las situaciones académicas vividas por los estudiantes de enfermería tienen potencial para el desarrollo de la autoestima inadecuada, lo que impacta en el estrés y en la mala calidad del sueño, así como en otros desenlaces negativos para la salud física y emocional^(26)^.

La comprensión de las relaciones entre antecedentes y consecuentes del diagnóstico fue esencial para construir la estructura teórica predictiva de las relaciones causales establecidas en estos componentes. La TAM autoestima inadecuada situacional en estudiantes de enfermería puede ayudar a la inferencia diagnóstica de los enfermeros en la atención a la salud estudiantil.

Entre las limitaciones del estudio se destacan la extracción de los conceptos clave (atributos, antecedentes y consecuentes) realizada únicamente por el investigador principal, sin la colaboración de otros investigadores, y el hecho de tratarse de un tema poco explorado, con escasa cantidad de investigaciones previas sobre el diagnóstico de autoestima inadecuada situacional, lo que demuestra que este trabajo cubre una laguna y sirve de base para futuras investigaciones que profundicen en la temática.

La TAM elaborada en este estudio puede contribuir a la práctica del enfermero al posibilitar la identificación, entre los factores presentados por el estudiante, de aquellos que probablemente originaron el diagnóstico. Esta información puede ofrecer subsidios para la elaboración de un plan de cuidados individualizado y eficiente centrado en los factores etiológicos sensibles a las intervenciones de enfermería.

Asimismo, el pictograma simboliza las relaciones de causalidad establecidas entre los antecedentes y los consecuentes del diagnóstico. Esta representación ayuda a verificar de forma eficaz la distribución de los indicadores clínicos en las dimensiones del diagnóstico, así como la jerarquía causal de los factores etiológicos.

## Conclusión

El mapeo de la producción científica en el contexto de los diagnósticos de enfermería sobre la autoestima inadecuada situacional en estudiantes de enfermería mostró una escasa exploración del tema tanto a nivel nacional como internacional.

La TAM desarrollada para el diagnóstico de enfermería autoestima inadecuada situacional en estudiantes de enfermería presentó cinco atributos, quince antecedentes y veintitrés consecuentes clínicos. Además, se elaboraron un pictograma ilustrativo, ocho proposiciones y doce relaciones causales y evidencias para la práctica, fundamentadas en el MAR, promoviendo la conexión entre teoría y práctica. Este enfoque permite traducir el conocimiento científico en acciones concretas, posibilitando una atención más holística y dirigida a los estudiantes de enfermería.

La construcción de la TAM posibilitó una comprensión profunda de las principales causas y consecuencias del diagnóstico de autoestima inadecuada situacional en estudiantes de enfermería. De este modo, los enfermeros disponen de herramientas validadas y basadas en evidencias para cualificar la atención a la salud de este público y fortalecer la Ciencia de la Enfermería.

Asimismo, se fomenta el desarrollo de futuros estudios orientados a la validación empírica de este diagnóstico, garantizando su aplicabilidad y eficacia en la práctica clínica.
